# SOX5 Orchestrates Malignant Evolution via Promoter‐Centric Chromatin Remodeling in MYC‐Driven B‐Cell Lymphoma

**DOI:** 10.1002/advs.76656

**Published:** 2026-07-17

**Authors:** Yiyou Mao, Zhenyuan Jia, Xiao‐Qin Mu, Ruilin Wang, Jianli Ma, Qingyuan Zhang, Yuwei Deng

**Affiliations:** ^1^ Department of Medical Oncology Harbin Medical University Cancer Hospital Harbin China; ^2^ Department of Genomics Genomics Research Center (Key Laboratory of Gut Microbiota and Pharmacogenomics of Heilongjiang Province) College of Pharmacy Harbin Medical University Harbin China; ^3^ Department of Pharmacy National Key Laboratory of Frigid Zone Cardiovascular Diseases Harbin Medical University Harbin China; ^4^ Department of Hematology and Endocrinology 962^ed^ Hospital of Joint Logistic Support Force of the Chinese People's Liberation Army China; ^5^ Department of Radiation Oncology Harbin Medical University Cancer Hospital Harbin China

**Keywords:** AAV6‐shRNA therapy, *MYC*‐driven B‐cell lymphoma, *PCNP*, promoter‐proximal accessibility, *SOX5*

## Abstract

Early events that bias germinal‐center B cells toward malignant evolution remain incompletely defined. Here, we uncover Sex‐determining Region Y Box 5 (*SOX5*) as an early‐stage epigenetic modifier in myelocytomatosis oncogene (*MYC*)‐driven B‐cell lymphoma. Single‐cell transcriptomics of lymph nodes from CRISPR/Cas9‐engineered *Myc^Cd19‐Cre^* B‐cell lymphoma chimeric mice resolve a Ki67–high proliferative compartment at an early stage with enriched *SOX5* transcripts. CRISPRa, CRISPR knockout, and multi‐omic profiling reveal a *SOX5*‐centered chromatin program defined by de novo promoter‐proximal occupancy coupled to contraction of promoter accessibility. Mechanistically, *SOX5* binding at the Proteolytic Signal‐Containing Nuclear Protein (*PCNP*) promoter represses its transcription, thereby reducing apoptosis and alleviating G2/M arrest. Consequently, B‐cell–targeted adeno‐associated virus 6 (AAV6)‐sh*SOX5* reduces Ki67‐positive *PAX5*‐positive malignant B cells and partially restores follicular architecture. Together, *SOX5*‐mediated, promoter‐centric repression of *PCNP* and other effectors installs and maintains an early‐stage proliferative state in *MYC*‐driven B‐cell lymphoma, which nominates *SOX5*‐directed intervention as a candidate therapeutic strategy for further investigation.

## Graphical abstract

In MYC‐enforced B‐cell lymphoma, SOX5 occupies promoter‐proximal regulatory regions and is associated with reduced chromatin accessibility at the PCNP locus. PCNP repression promotes proliferative remodeling by limiting apoptosis and cell‐cycle restraint. B‐cell‐targeted AAV6‐shSOX5 relieves this SOX5–PCNP regulatory axis and attenuates lymphoma‐associated B‐cell expansion, demonstrating the therapeutic potential of AAV6‐mediated SOX5‐targeted gene therapy in B‐cell lymphoma.

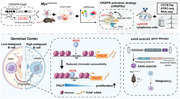

## 1 Introduction

Germinal centers (GCs) constitute specialized immune niches where antigen‐experienced B cells undergo clonal expansion, somatic hypermutation of immunoglobulin loci, and affinity maturation to generate high‐affinity antibody repertoires [1, 2]. The molecular machinery driving robust proliferation and mutagenesis within GCs carries intrinsic oncogenic risk; once cell cycle and apoptotic checkpoints fail, these physiological programs get co‐opted to fuel B‐cell malignant transformation. Diffuse large B‐cell lymphoma (DLBCL) and follicular lymphoma (FL) represent the two most prevalent subtypes of B‐cell non‐Hodgkin lymphoma [3]. Clinically, a substantial proportion of FL patients experience rapid disease progression within two years following diagnosis or frontline immunochemotherapy, while close to half of DLBCL patients develop relapsed or refractory disease after initial standard‐of‐care regimens [4, 5]. Such poor clinical outcomes highlight an urgent unmet need to dissect GC‐intrinsic regulatory circuits that interface with therapeutic responsiveness, stromal microenvironment cues, intratumoral clonal heterogeneity, and longitudinal tumor evolutionary trajectories to shape aggressive lymphoma phenotypes [6].

Tumor progression and intratumoral heterogeneity are universally accompanied by profound transcriptomic and epigenetic remodeling events [7, 8]. Building on our prior clinical work profiling HBsAg‐positive POD24 FL specimens, we engineered a MYC^Cd19‐Cre^ chimeric mouse model with conditional *MYC* overexpression that faithfully recapitulates the aberrant hyperproliferative signature of germinal center B cells. Given the high frequency of *MYC* translocations and amplifications in high‐grade B‐cell malignancies [9], this genetically defined model offers a tractable experimental platform to dissect *MYC*‐driven proliferative rewiring in GC B cells. Combined with our earlier spatial transcriptomic analyses of early‐relapse FL and mechanistic investigations into HBV‐associated lymphomagenesis, this paired clinical and preclinical framework enables systematic interrogation of molecular programs underpinning aggressive B‐cell states [10, 11, 12].

Members of the SOX transcription factor superfamily function as master arbiters of cellular identity, largely via their capacity to remodel genome‐wide chromatin accessibility and rewire lineage‐specific transcriptional cascades [13, 14]. *SOX5*, one well‐characterized SOX paralog, preferentially engages H3K27ac‐marked enhancer regions to redirect transcriptional output, with established roles in neural differentiation, cellular senescence, and multiple solid tumor pathologies [15, 16]. Though recurrent *SOX5* gene fusions including P2RY8::SOX5 and IGH‐SOX5 rearrangements have been documented in rare FL subsets, these genomic alterations only confirm *SOX5* dysregulation in a minor fraction of B‐cell lymphomas [17, 18]. Critically, existing literature fails to resolve whether wild‐type, non‐rearranged *SOX5* exerts functional oncogenic effects to sustain the hyperproliferative cell states imposed by constitutive *MYC* signaling.

In the present study, we integrated CRISPR‐mediated transcriptional activation (CRISPRa) with multi‐layered omics profiling and cellular functional assays to dissect SOX5's mechanistic contributions to GC B‐cell malignant reprogramming. Our multi‐omic analyses pinpoint de novo *SOX5* occupancy proximal to the transcription start site of *PCNP* (Proteolytic Signal‐Containing Nuclear Protein), an epigenetic event coupled with suppressed *PCNP* transcription and sustained lymphoma cell proliferation. In vivo therapeutic intervention using CD19‐targeted AAV6‐delivered shRNA against *SOX5* efficiently depleted Ki67^+^ proliferative malignant B‐cell populations and partially restored disrupted follicular tissue architecture. Collectively, our findings nominate the *SOX5*–*PCNP* regulatory axis as a druggable epigenetic node for developing targeted anti‐lymphoma strategies.

## 2 Results

### 2.1 *SOX5* is Identified as a Key Regulator Enriched in the Proliferative Early State of *MYC*‐Driven B‐Cell Malignancy

**FIGURE 1:**
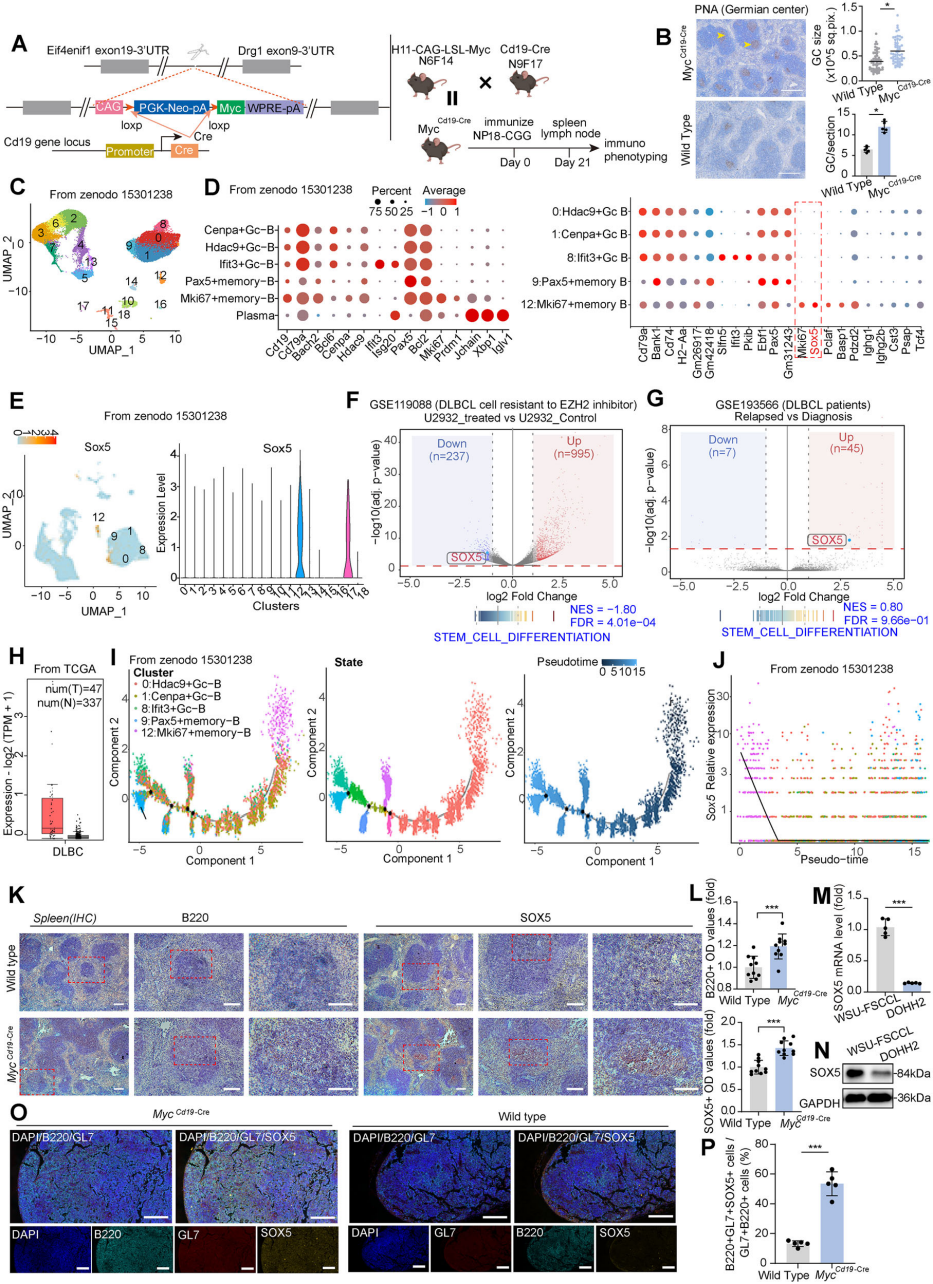
*SOX5* as a key regulator of early *MYC*‐driven hyper‐proliferation in malignant B cells. (A) Construction strategy of chimeric mice regarding *Myc*‐overexpression by CRISPR/Cas9‐mediated homologous recombination and then recombined by CD19‐promoter‐Cre. (B) Immunohistochemical staining of the germinal center after immunization in *Myc^Cd19‐Cre^* mice. Scale bar, 100 µm. (C) UMAP illustrated 0‐18 clusters based on single‐cell sequencing (Zenodo 15301238). (D) Bubble diagram demonstrated the marker genes in B‐cell subclusters. (E) UMAP analysis and violin plot showed the *SOX5* expression among B‐cell clusters. UMAPs in panels C and E were generated as separate embeddings using different PCA dimensions as input to UMAP construction: panel C used dims = 1:20, whereas panel E used dims = 1:30. (F,G) Volcano plots (top) showing differential gene expression and hallmark gene‐set enrichment (bottom) for pathways associated with *SOX5* in GSE119088 (F) and GSE198713 (G). (H) Expression of *SOX5* in human large B‐cell lymphoma compared with adjacent normal tissues based on TCGA dataset. (I) Pseudo‐time trajectory plot of cell clusters (left). The trajectories of different cell states (State 1–6) in the proposed chronological analysis (right). Data from zendo 15301238. (J) The distribution of cells in the proposed chronology, with color shades indicating the change in the cells’ pseudo‐time from early to late (left). A scatter plot of gene expression vs. pseudo‐time showed the expression trend of *SOX5* genes on pseudo‐time, with colors indicating different cell clusters (right). Data from zendo 15301238. (K) Immunohistochemical staining showed the *SOX5* expression in B220+ B cells in *Myc^Cd19‐Cre^* mice compared with wild type. Scale bar, 100 µm. (L) Quantification of staining intensity of K. (M) mRNA expression of *SOX5* in B‐cell lymphoma cell lines using qRT‐PCR. mean fold‐change mRNA levels normalized to Gapdh, ± SEM; unpaired *t*‐test, **p < 0.01, ***p < 0.001. (N) Immunoblots showed the protein expression of *SOX5* in B‐cell lymphoma cell lines. (O) Multiple immunofluorescence assays revealed the expression of *SOX5* in germinal center B cells (B220+GL7+) in *Myc^Cd19‐Cre^* mice compared with wild type. Scale bar, 100 µm. (P) Quantification of staining intensity for cell percentage of O.

Given the pivotal role of *MYC* oncogene in aggressive B‐cell lymphoma [19, 20], we generated an H11‐CAG‐LSL‐Myc knock‐in chimeric mouse using CRISPR/Cas9‐mediated homologous recombination at the Hipp11 locus (Figure 1A and Figure S1A). Correct H11‐CAG‐LSL‐Myc insertion and germline transmission were confirmed by F1 5′/3′ homology‐arm PCR and sequencing, with F2/F3 validation (Figure S1B) Crossing these mice with CD19‐Cre mice enabled B cell–specific recombination, generating Myc^Cd19‐Cre^ offspring in which *Myc* overexpression in the CD19‐positive B‐cell nuclear compartment led to GC amplification (PNA‐marked regions) (Figure 1B) and an abnormal proliferative B‐cell phenotype, as previously described [12], including increased tumor burden and histopathological abnormalities (Figure S1C,D), and cellular Myc activation was verified at transcription, and protein level in lymph‐node B220‐positive B cells using FACS, as well as spatial strong signal of nuclear Myc within B220‐marked B cells (Figure S1E–H).

Single‐cell transcriptomic profiling of lymph nodes from Myc^Cd19‐Cre^ mice was first analyzed at the whole‐sample level, after which B‐cell clusters were re‐embedded for focused analysis (Zenodo 10.5281/zenodo.15301238) [12]. This analysis resolved 19 clusters, including a proliferative Mki67+ B‐cell compartment (cluster 12) (Figure 1C and Figure S2A). Based on differentiation signatures across B‐cell subtypes, cluster 12 was conservatively annotated as a proliferative memory‐like B‐cell compartment, retaining B‐cell identity markers (*Cd74, Cd79a, Pax5*) together with *Bach2, Prdm1*, and *Bcl2* expression (Figure 1D). Cluster 12 was further characterized by the top five upregulated genes—*Mki67*, *SOX5*, *Pclaf*, *Basp1*, and *Pdzd2*—and differential expression analysis ranked *SOX5* among the most upregulated transcripts alongside effectors of proliferation and DNA replication (*Pclaf, Pola1, Mybl1*, et al; Figure 1D,E and Table S1). The effect size for *SOX5* was robust across analytic approaches. Consistently, *SOX5* upregulation within this compartment aligned with the Mki67‐marked proliferative status of early *Myc*‐overexpressed B cells (Figure S2B), but not affected the long‐term survival of mice (Figure S2C).

Interrogation of independent cohorts (GSE119088, GSE193566) confirmed *SOX5* upregulation in progressive tumors (resistant DLBCL cell lines, relapsed DLBCL patients, respectively), mirroring our scRNA‐seq findings and linking to pathways of stem cell differentiation (evolution process) (Figure 1F,G and Figure S2D). *SOX5* expression in human Lymphoid Neoplasm Diffuse Large B‐cell Lymphoma (DLBC) was relatively elevated compared with normal controls (GEPIA2, TCGA/GTEx, tumor = 47, Num = 337; Figure 1H), and was negatively correlated with PFS (Figure S2E). Exploratory pseudotime ordering (Monocle2) placed the proliferative cluster near an early branch of the inferred trajectory and showed a gradual decline in *SOX5* expression along the inferred axis (Figure 1I,J). Its higher expression in early‐state lymphoma cells (Figure S2F), together with enrichment of differentiation/evolution programs (Figure S2G), suggests that subsequent *SOX5* downregulation precedes cell‐cycle exit and further maturation. Reference‐based label transfer using the wild‐type group from GSE224782 as an annotated normal mouse reference assigned cluster 12 mainly to Early_Centroblast, Centroblast, Transitioning_CB_CC, Centrocyte, and Prememory states, supporting its placement along a GC‐to‐prememory B‐cell transition continuum in the Myc^Cd19‐Cre^ model (Figure S2H–J).

Immunohistochemistry revealed a significant overall increase of *SOX5* protein in B220^+^ follicles of *Myc^Cd19‐Cre^* spleens relative to wild‐type (H‐score, positive area; Figure 1K,L). We next assessed *SOX5* expression in two FL‐derived B‐cell lymphoma lines to select a suitable perturbation model. WSU‐FSCCL (slowly progressing) [21] showed detectable *SOX5* expression and was therefore used for subsequent *SOX5* depletion and CRISPRa‐based perturbation assays (Figure 1M,N). DOHH2 (aggressive B‐cell features) [22] was subsequently used as an additional lymphoma‐cell context for validation experiments. Consistent with this, confined within the germinal center (B220+GL7+), higher proportion of *SOX5*+ GCB were further observed in lymph node of *Myc^Cd19‐Cre^* than wild‐type (Figure 1O,P).

Collectively, integration of single‐cell clustering, independent cohorts, and in situ tissue validation establishes *SOX5* as a candidate regulator enriched in a MYC‐enforced proliferative B‐cell compartment.

### 2.2 *SOX5* Promotes a Malignant Proliferation Phenotype in B‐Cell Lymphoma

**FIGURE 2:**
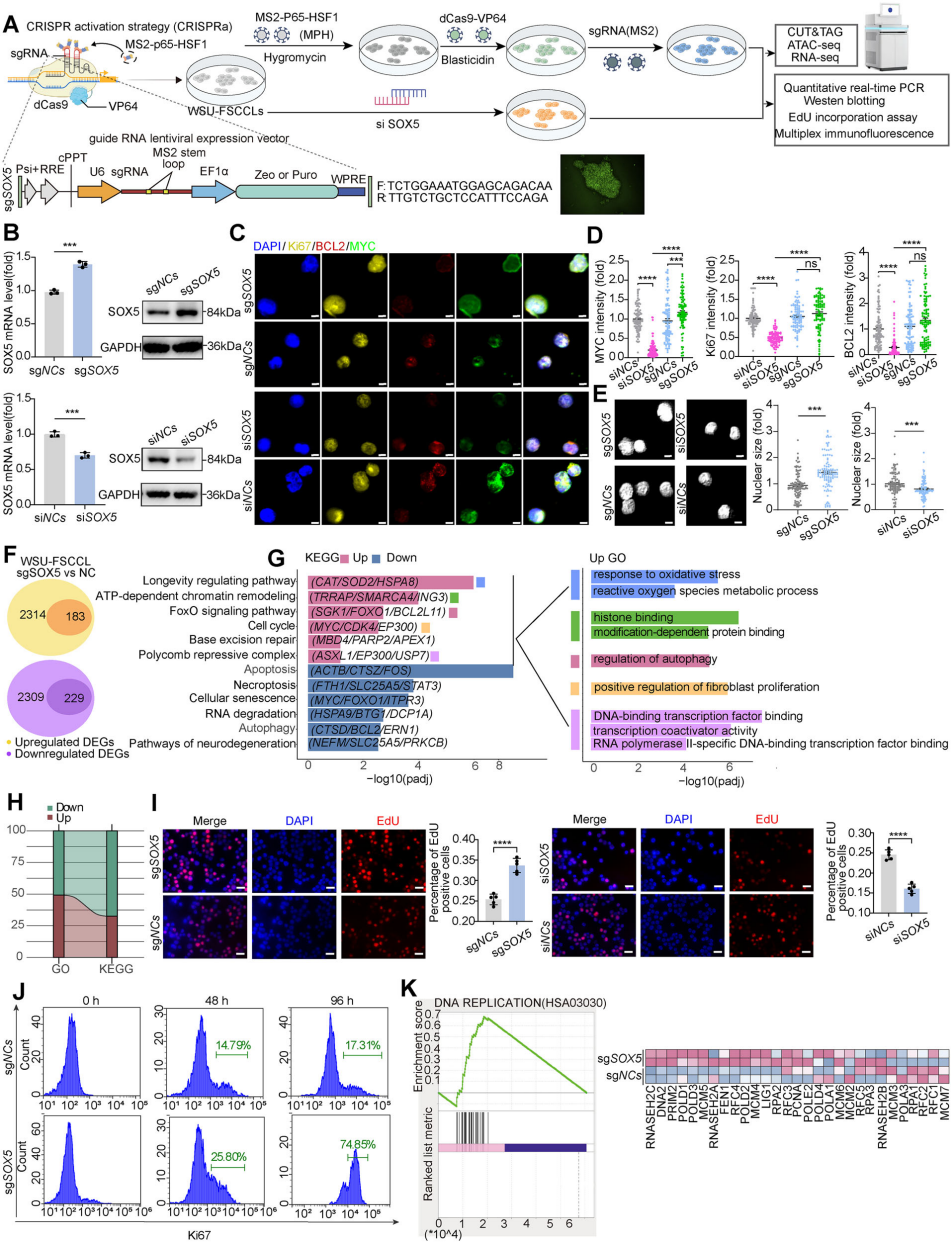
*SOX5* promotes a malignant proliferation phenotype in B‐cell lymphoma. (A) CRISPR activation strategy (CRISPRa) of *SOX5* (sg*SOX5*) and the overall schematic diagrams for the study. (B) qRT‐PCR analyzed mRNA expression of *SOX5* in B‐cell lymphoma cell lines after sg*SOX5* (above) or *SOX5* knockdown (si*SOX5*, below). mean fold‐change mRNA levels normalized to Gapdh, ± SEM; unpaired t test, **p < 0.01, ***p < 0.001 (left). Immunoblots showed the protein expression of *SOX5* (right). (C) Multiplex immunofluorescence showed the Ki67, BCL2 and *MYC* expression in cell lines transduced with sg*SOX5* / transfected with si*SOX5*. Scale bar, 5 µm. (D) Quantification of the intensity of (C). SiNC as reference. (E) Representative images of the nuclear changes and quantifications analysis in cell lines transferred with sg*SOX5* or si*SOX5*. Scale bar, 5 µm. siNC as reference. (F) Venn diagrams showing DEGs in sg*SOX5* vs. control cells. Inner sectors indicate annotated functional subsets: cell‐cycle‐related genes among upregulated DEGs and apoptosis‐related genes among downregulated DEGs. (G) Bar plot showing enriched KEGG pathways with the corresponding genes (left) and enriched GO terms (right). (H) Trend consistency analysis of GO and KEGG. (I) Representative images of 5‐Ethynyl‐2'‐deoxyuridine (EdU) staining and corresponding quantification analysis showed the proliferation status in cell lines transferred with sg*SOX5* or si*SOX5*. Scale bar, 15 µm. (J) Flow cytometric analysis of Ki67 in cell lines following transferred with sg*SOX5*. (K) The enrichment scores of DNA replication pathways (above) and corresponding differential expression genes (below) in cell lines following transferred with sg*SOX5*. For microscopy‐based quantification, multiple fields were averaged within each independent experiment before statistical comparison. For qRT‐PCR and flow cytometry, *n* denotes independent biological replicates. Significance is denoted *p < 0.05, **p < 0.01, ***p < 0.001, ****p < 0.0001.

We used a CRISPR‐based transcriptional activation approach (hereafter CRISPRa) [23] to upregulate endogenous *SOX5*. As documented by studies of HMG‐domain mutants such as *SOX5*N561H and *SOX5*R571W, *SOX5* possesses sequence‐specific DNA‐binding capacity [24]. Our sgRNA sequence (sg*SOX5*) was adopted from a previously published study [25], with transfection efficiency confirmed by a GFP reporter (Figure 2A), alongside siRNA‐based knockdown in the primary B‐cell lymphoma line (Figure 2B). si*SOX5* reduced Ki67 expression together with BCL2 and *MYC* levels, whereas sg*SOX5* increased the expression of these proliferation‐related genes (Figure 2C,D). The sg*SOX5* cells also exhibited enlarged nuclei and a pro‐proliferative morphology (Figure 2E).

Transcriptomic profiling of sg*SOX5* cells revealed a distinct principal component distribution compared to wild‐type cells (Figure S3A). RNA‐seq identified 2,314 upregulated and 2,309 downregulated genes in sg*SOX5* cells relative to controls. Among these, 183 upregulated genes were annotated to cell‐cycle‐related programs, whereas 229 downregulated genes were annotated to apoptosis‐related programs (Figure 2F and Figure S3B). Accordingly, sg*SOX5* robustly upregulated a gene set enriched in ATP‐dependent chromatin remodeling and cell cycle, while downregulating processes enriched in apoptosis and necroptosis (Figure 2G). Integrated GO and KEGG analyses highlighted significant enrichment in cell proliferation activities (metabolic process, modification, transcription) (Figure 2G,H). We further confirmed the accelerated cell proliferation phenotype with sg*SOX5* (Figure 2I,J), consistent with activation of DNA‐replication programs identified in the transcriptome (Figure 2K) and a shift away from the G1 phase (Figure S3C). Together, these data support a functional role for *SOX5* in promoting lymphoma‐cell proliferation and motivated chromatin‐level mapping to identify candidate downstream regulatory targets.

### 2.3 *SOX5* Activation Links Genome‐Wide Occupancy to Promoter‐Proximal Accessibility Contraction

**FIGURE 3:**
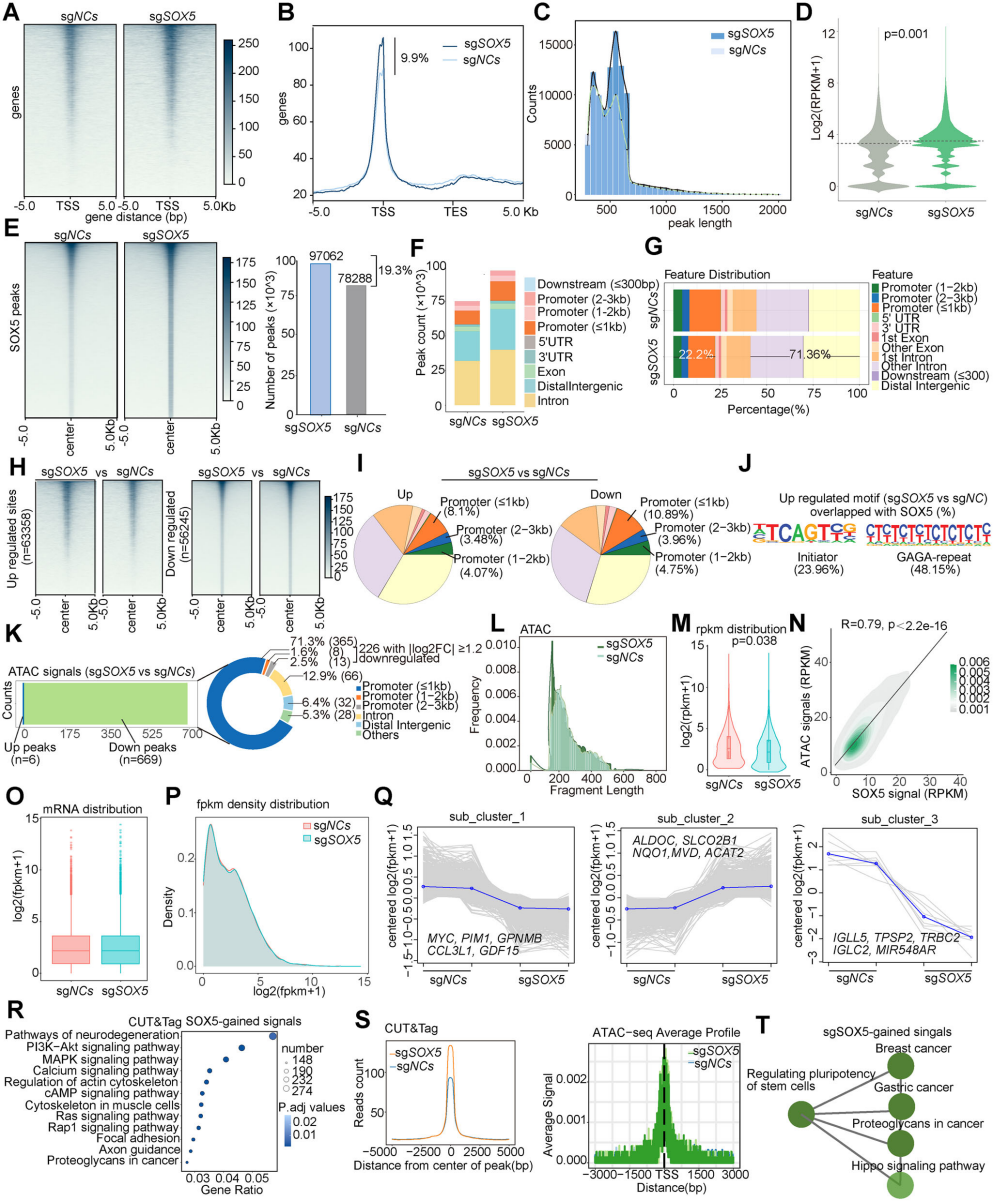
*SOX5* activation rewires chromatin through de novo promoter‐proximal binding and global contraction of promoter accessibility. (A) Heatmap showing *SOX5* CUT&Tag signals near genes in WSU‐FSCCL cells transduced with sg*SOX5*. Additional CUT&Tag quality‐control analysis showed a higher FRiP score in the SOX5 CUT&Tag sample than in the control sample (16.08% vs. 11.29%). (B) Gene‐proximal signal distribution corresponding to (A). (C) Distribution of peak lengths in the two groups. (D) Violin plot showing the CUT&Tag signals at *SOX5*‐gained promoters in cell lines transduced with sg*SOX5*. (E) Heatmap showed the *SOX5* CUT&Tag sequencing signals in WSU‐FSCCL cell lines transduced with sg*SOX5*. Column diagrams showed the gained number of peaks. (F) Bar chart showing peak numbers across genomic functional regions in WSU‐FSCCL cell lines transduced with sg*SOX5* and control. (G) Distribution of annotated peak regions. (H) Heatmap showed the upregulated and downregulated *SOX5*‐binding signals from *SOX5* CUT&Tag sequencing in cell lines transduced with sg*SOX5* compared to control. (I) Pie chart showing the genomic distribution of differential peaks in (H). (J) Representative enriched motif associated with *SOX5*‐gained signals. (K) Differential ATAC‐seq peaks under sg*SOX5* vs. control (FDR < 0.05): down, *n* = 669; up, *n* = 6. Among significant annotated ATAC peaks, promoter‐class sites dominate (counts shown). (L) Fragment‐length distribution of ATAC‐seq reads in sg*SOX5* and control group. (M) The ATAC average signals under sg*SOX5* vs. control (Student's *t*‐test, *p* = 0.038). (N) Correlation between *SOX5* and ATAC signals on *SOX5*‐binding sites. (O) RNA‐seq modules illustrate limited transcriptional shifts between sg*SOX5* and control groups. (P) Transcriptional density distribution of sg*SOX5* and control groups. (Q) The three subclusters of differentially expressed genes under sg*SOX5* vs. control based on the coordinated transcriptional shifts identified by hierarchical clustering. (R) KEGG enrichment of loci/genes linked to sg*SOX5*‐gained regulation; adjusted *p* values are shown in panel. (S) The peak signals distribution in CUT&Tag‐seq and ATAC‐seq. (T) Summary of the enriched tumor‐associated pathway crosstalk network based on sg*SOX5*‐gained signals.

We first examined the genome‐wide *SOX5* occupancy landscape by CUT&Tag before focusing on promoter‐proximal regulatory events. Across the genome, *SOX5* signals were intensively distributed across transcriptional start site covering promoter‐associated regions (Figure 3A). Approximately 9.9% of genes showed expanded *SOX5*‐associated signal after *SOX5* activation (Figure 3B). *SOX5* activation markedly increased the number and intensity of binding peaks across genomic segments (Figure 3C,D). After quality‐control filtering, we identified a subset of *SOX5*‐combined sites gained after *SOX5* activation (Figure 3E and Figure S3D). The overall *SOX5* occupancy landscape was dominated by distal elements and introns (∼71.36%), with promoter‐associated peaks comprising ∼22.2%, consistent with the broad regulatory distribution reported for *SOX5* in other contexts [25] (Figure 3F,G and Table S2). Having established this genome‐wide distribution, we next asked whether *SOX5* activation preferentially altered specific classes of regulatory elements. In contrast to the broad distribution of total *SOX5* peaks, differential *SOX5*‐associated signals after sg*SOX5* activation showed a relative enrichment in promoter‐proximal regions, particularly within 1 kb of annotated TSSs (Figure 3H,I and Figure S3E). *SOX5* peaks were close to H3K4me3 promoter peaks from other B‐cell lymphomas (ENCSR052WRV) (*p* = 0.05), further supporting enhanced promoter signals (Figure S3F). Motif analysis of the retained sgSOX5‐gained peaks recovered SOX‐family motifs together with promoter‐associated and GAGA/Polycomb‐linked regulatory motifs (Figure 3J and Figure S3G,H). Especially, we identified a de novo sgSOX5‐gained motif whose reverse‐complement sequence aligned with reported SOX5/SOX‐family PWMs, similar to Aef1 (enhancer factor in fly) with transcript activation (Figure S3I). These motif features suggested that a subset of *SOX5*‐associated sites may reside within a transcription‐regulatory context (Table 1).

**TABLE 1:** Homer known Motif enrichment results (sg*SOX5*‐vs‐control/Motif/up).

Rank	Motif	Name	*p*‐ value	log *p*‐ value	q‐value (Benjamini)	# target sequences with Motif	% of targets sequences with Motif	# background sequences with Motif	% of background sequences with Motif
1	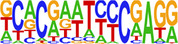	AIL7(AP2EREBP)/colamp‐AIL7‐DAP‐Seq(GSE60143)/Homer	1e‐3	−9.106e+00	0.1117	132.0	4.40%	1478.5	3.14%
2	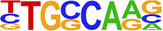	NF1‐halfsite(CTF)/LNCaP‐NF1‐ChIP‐Seq(Unpublished)/Homer	1e‐3	−7.658e+00	0.2375	583.0	19.43%	8043.9	17.10%
3	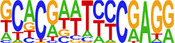	PLT1(AP2EREBP)/colamp‐PLT1‐DAP‐Seq(GSE60143)/Homer	1e‐3	−7.512e+00	0.2375	22.0	0.73%	154.6	0.33%
4	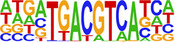	TGA5(bZIP)/col‐TGA5‐DAP‐Seq(GSE60143)/Homer	1e‐3	−6.993e+00	0.2375	23.0	0.77%	171.6	0.36%
5	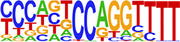	PRDM15(Zf)/ESC‐Prdm15‐ChIP‐Seq(GSE73694)/Homer	1e‐2	−6.611e+00	0.2706	321.0	10.70%	4266.6	9.07%
6	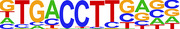	ERRg(NR)/Kidney‐ESRRG‐ChIP‐Seq(GSE104905)/Homer	1e‐2	−6.465e+00	0.2706	262.0	8.73%	3422.5	7.28%
7	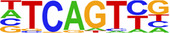	Initiator/Drosophila‐Promoters/Homer	1e‐2	−6.455e+00	0.2706	675.0	22.49%	9539.8	20.28%
8	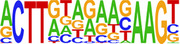	NTM1(NAC)/col‐NTM1‐DAP‐Seq(GSE60143)/Homer	1e‐2	−6.379e+00	0.2706	130.0	4.33%	1561.3	3.32%
9	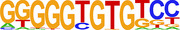	KLF10(Zf)/HEK293‐KLF10.GFP‐ChIP‐Seq(GSE58341)/Homer	1e‐2	−6.360e+00	0.2706	206.0	6.86%	2627.4	5.59%
10	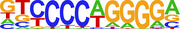	EBF1(EBF)/Near‐E2A‐ChIP‐Seq(GSE21512)/Homer	1e‐2	−6.011e+00	0.2706	382.0	12.73%	5207.7	11.07%
11	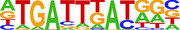	PBX2(Homeobox)/K562‐PBX2‐ChIP‐Seq(Encode)/Homer	1e‐2	−5.947e+00	0.2706	265.0	8.83%	3501.9	7.45%
12	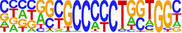	BORIS(Zf)/K562‐CTCFL‐ChIP‐Seq(GSE32465)/Homer	1e‐2	−5.928e+00	0.2706	62.0	2.07%	665.7	1.42%
13	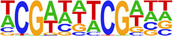	AT5G22990(C2H2)/col‐AT5G22990‐DAP‐Seq(GSE60143)/Homer	1e‐2	−5.794e+00	0.2706	17.0	0.57%	123.3	0.26%
14	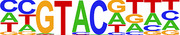	SPL1(SBP)/colamp‐SPL1‐DAP‐Seq(GSE60143)/Homer	1e‐2	−5.617e+00	0.2706	463.0	15.43%	6444.7	13.70%
15	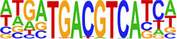	TGA3(bZIP)/colamp‐TGA3‐DAP‐Seq(GSE60143)/Homer	1e‐2	−5.541e+00	0.2706	14.0	0.47%	95.7	0.20%
16	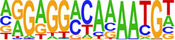	ZNF675(Zf)/HEK293‐ZNF675.GFP‐ChIP‐Seq(GSE58341)/Homer	1e‐2	−5.520e+00	0.2706	53.0	1.77%	561.3	1.19%
17	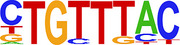	Foxo1(Forkhead)/RAW‐Foxo1‐ChIP‐Seq(Fan_et_al.)/Homer	1e‐2	−5.307e+00	0.2932	583.0	19.43%	8276.1	17.60%
18	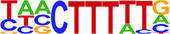	dof24(C2C2dof)/col‐dof24‐DAP‐Seq(GSE60143)/Homer	1e‐2	−5.246e+00	0.2945	673.0	22.43%	9644.0	20.51%
19	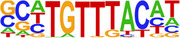	FOXK2(Forkhead)/U2OS‐FOXK2‐ChIP‐Seq(E‐MTAB‐2204)/Homer	1e‐2	−5.211e+00	0.2945	199.0	6.63%	2599.3	5.53%
20	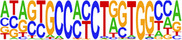	CTCF(Zf)/CD4+‐CTCF‐ChIP‐Seq(Barski_et_al.)/Homer	1e‐2	−5.032e+00	0.3282	37.0	1.23%	371.8	0.79%
21	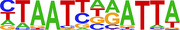	Phox2a(Homeobox)/Neuron‐Phox2a‐ChIP‐Seq(GSE31456)/Homer	1e‐2	−4.907e+00	0.3541	142.0	4.73%	1804.7	3.84%
22	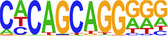	Unknown‐ESC‐element(?)/mES‐Nanog‐ChIP‐Seq(GSE11724)/Homer	1e‐2	−4.902e+00	0.3541	226.0	7.53%	3011.5	6.40%
23	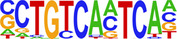	Pbx3(Homeobox)/GM12878‐PBX3‐ChIP‐Seq(GSE32465)/Homer	1e‐2	−4.897e+00	0.3541	74.0	2.47%	860.7	1.83%
24	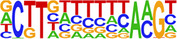	VND6(NAC)/col‐VND6‐DAP‐Seq(GSE60143)/Homer	1e‐2	−4.824e+00	0.3541	230.0	7.66%	3075.4	6.54%
25	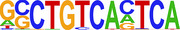	PBX1(Homeobox)/MCF7‐PBX1‐ChIP‐Seq(GSE28007)/Homer	1e‐2	−4.762e+00	0.3541	27.0	0.90%	254.6	0.54%
26	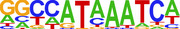	HOXA9(Homeobox)/HSC‐Hoxa9‐ChIP‐Seq(GSE33509)/Homer	1e‐2	−4.742e+00	0.3541	211.0	7.03%	2805.7	5.97%
27	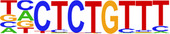	REF6(Zf)/Arabidopsis‐REF6‐ChIP‐Seq(GSE106942)/Homer	1e‐2	−4.726e+00	0.3541	194.0	6.46%	2560.5	5.44%
28	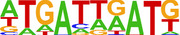	LIN‐39(Homeobox)/cElegans.L3‐LIN39‐ChIP‐Seq(modEncode)/Homer	1e‐2	−4.700e+00	0.3541	381.0	12.70%	5312.8	11.30%
29	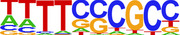	DEL2(E2FDP)/col‐DEL2‐DAP‐Seq(GSE60143)/Homer	1e‐2	−4.693e+00	0.3541	83.0	2.77%	991.4	2.11%
30	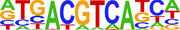	TGA4(bZIP)/colamp‐TGA4‐DAP‐Seq(GSE60143)/Homer	1e‐2	−4.677e+00	0.3541	62.0	2.07%	707.7	1.50%
31	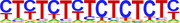	GAGA‐repeat/SacCer‐Promoters/Homer	1e‐2	−4.654e+00	0.3541	1366.0	45.52%	20400.6	43.38%
32	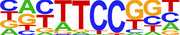	Fli1(ETS)/CD8‐FLI‐ChIP‐Seq(GSE20898)/Homer	1e‐2	−4.638e+00	0.3541	303.0	10.10%	4161.9	8.85%

*Note*: Total target sequences = 3000, Total background sequences = 47086.

Based on this shift from a broad genome‐wide occupancy landscape to promoter‐proximal differential events, we next asked whether *SOX5*‐associated promoter regions showed coordinated changes in chromatin accessibility. Therefore, ATAC‐seq under sg*SOX5* conditions identified 675 significant differential peaks (FDR<0.05), of which 669 decreased and 6 increased (Figure 3K, left). Within the annotated significant peaks (*n* = 512), ∼75.4% localized to promoters (≤1 kb: 365; 1–2 kb: 8; 2–3 kb: 13). Strikingly, all 226 promoter‐class peaks with |log2FC| ≥ 1.2 exhibited decreased accessibility, indicating widespread promoter‐proximal accessibility contraction upon *SOX5* activation (Figure 3K, right). The marked reduction in ATAC signals in sg*SOX5* cells (Figure 3L,M and Figure S3J) correlated with gains in *SOX5* binding (Figure 3N) and corresponded to weakened open chromatin near TSSs (Figure S3K). This inverse relationship between *SOX5* gain and ATAC loss argues against a simple open‐chromatin artifact as the sole explanation for the retained *SOX5* CUT&Tag signal. Key examples of promoter closure included *RPE, SGK1, SMARCA2*, and *PCNP* (Figure S3L). Despite this widespread closing, FPKM values changed only modestly for most affected genes (Figure 3O,P). Nevertheless, coherent biological shifts emerged: downregulation of a pro‐senescence cluster (Cluster 1, Table S3), increase of a metabolic‐enzyme cluster (Cluster 2, Table S4), and downregulation of an immunomodulatory cluster (Cluster 3, Table S5), supporting a proliferative/growth‐conducive state (Figure 3Q).

Gained CUT&Tag peaks were enriched for cancer‐associated pathways including PI3K‐Akt, MAPK, cAMP, and Ras (Figure 3R), whereas gained ATAC peaks highlighted cell cycle and meiosis (Figure S3M). Notably, both CUT&Tag and ATAC‐seq signals increased in the proximal 2–3 kb region upstream of TSSs with *SOX5* occupancy at these cis‐regulatory elements (Figure 3S), underscoring *SOX5*‐mediated selective intervention in oncogenic and stemness pathways networks analyzed by visNetwork (Figure 3T). Together, these analyses move from the genome‐wide *SOX5* occupancy landscape to a subset of promoter‐proximal regulatory events in which gained *SOX5* signal is coupled to reduced chromatin accessibility, providing a framework for prioritizing candidate target genes.

### 2.4 *PCNP* is Prioritized as a Promoter‐Proximal Repressive Target of *SOX5*

**FIGURE 4:**
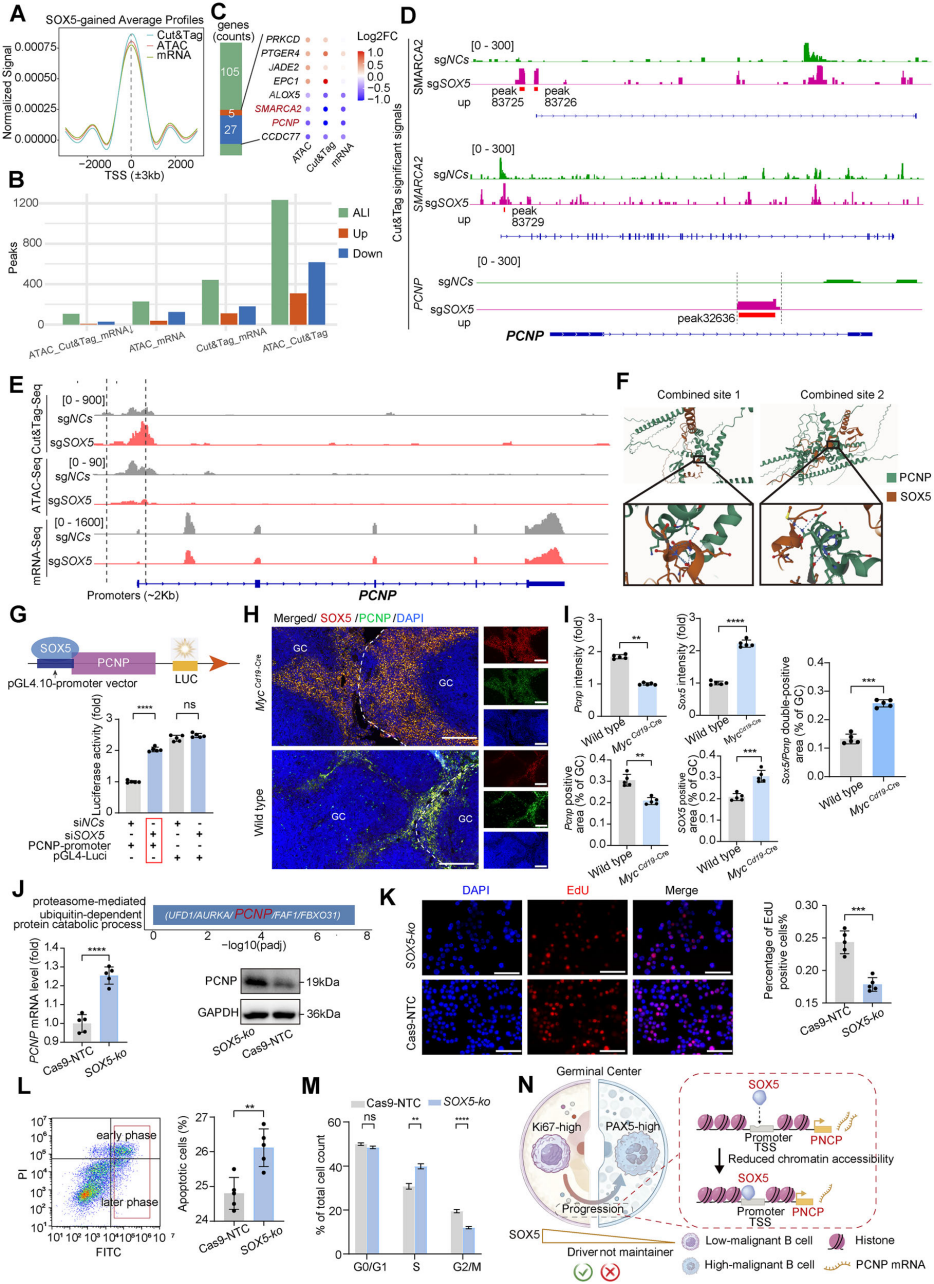
*SOX5* binds the *PCNP* promoter with promoter accessibility contraction, resulting in proliferative dysregulation. (A) TSS‐centered average profiles (±3 kb) for sg*SOX5*‐gained signals across CUT&Tag, ATAC‐seq, and RNA‐seq (sg*SOX5* vs. NC). (B) Tripartite concordance analysis among CUT&Tag, ATAC‐seq, and RNA‐seq, summarized by counts for All/Up/Down peaks. (C) Bubble graph showed the top‐ranked differential genes enriched based on the common peak signals among CUT&Tag, ATAC‐seq, and RNA‐seq. (D) IGV tracks showing signal peaks at the SMARCA2 and PCNP loci based on CUT&Tag. (E) Snapshot showing the binding pattern of *SOX5* and ATAC signals on the promoter of *PCNP* and the transcription of *PCNP* in *SOX5*‐overexpressing cell lines. (F) Predicted structures and detailed binding sites of *SOX5*–*PCNP* protein complex using AlphaFold. (G) Schematic of the *PCNP* promoter (∼2 kb) and reporter design. A promoter fragment centered on the CUT&Tag summit was cloned into pGL4.10 to assess transcriptional output under *SOX5* perturbation. Luciferase reporter assay for the *PCNP* promoter under si*SOX5* vs. siNC; *SOX5* knockdown significantly increases reporter activity. (H) Merged *SOX5*/*PCNP*/DAPI images and spatial quantification showing higher nuclear *SOX5* with lower *PCNP* in germinal centers of *Myc^Cd19‐Cre^* mice. Scale bar, 100 µm. (I) Box plots summarize *SOX5*‐positive area, *PCNP*‐positive area, double‐positive area, and intensity, with significance indicated. (J) *PCNP* upregulation upon *SOX5‐ko* at mRNA (fold) level using qRT‐PCR and immunoblot of *PCNP* with GAPDH as loading control. (K) Representative images and quantification of EdU‐positive cells of *SOX5‐ko* compared with Cas9‐NTC. Scale bar, 100 µm. (L,M) Cell death and cell‐cycle effects of *SOX5‐ko*. (L): Annexin V/PI flow plots and bar graph of apoptotic cells (%). (M): PI‐based cell‐cycle histograms and phase distribution (% of total cells). (N) The mechanism illustration: In early stage of malignancy, *SOX5* acts as a driver rather than a maintainer by enforcing promoter‐centric repression—exemplified at the *PCNP* locus—via de novo proximal occupancy and reduced chromatin accessibility, sustaining a Ki‐67‐positive proliferative state. Comparisons employed two‐sided tests; significance is denoted *p < 0.05, **p < 0.01, ***p < 0.001, ****p < 0.0001.

To define downstream effector axes of *SOX5*, we applied a tripartite concordance strategy. First, sg*SOX5* CUT&Tag profiles prioritized loci with gained *SOX5* motif‐containing promoters, supported by enrichment of SOX‐family motifs near peak summits. Second, we assessed ATAC‐seq changes at the same coordinates to classify increased vs. repressive/non‐activating accessibility. Third, we required transcriptional changes of the corresponding genes to be concordant with the nature of *SOX5* occupancy (Figure 4A,B and Figure S4A). This funnel‐like screen—consistent with the predominantly non‐opening ATAC‐seq landscape—nominated two high‐confidence candidates, *SMARCA2* and *PCNP* (Figure 4C). Chromatin‐accessibility dynamics showed a tighter correspondence between *SOX5* and *PCNP* (Figure S4B). IGV views showed TSS‐proximal binding at both loci, especially with a de novo *SOX5* peak at the *PCNP* promoter which was absent in controls (Figure 4D).

A coherent repressive pattern emerged at the PCNP locus. The PCNP promoter‐proximal peak was retained after CUT&Tag quality filtering and contained a nearby SOX‐family motif (Figure S4C,D). At the same coordinates, this retained *SOX5*‐associated peak coincided with reduced ATAC accessibility and decreased PCNP transcript levels, supporting its prioritization for reporter validation (Figure 4E). In silico structural modeling further supported a plausible *SOX5*–DNA interface consistent with promoter recognition (Figure 4F). A *PCNP* promoter fragment centered on the CUT&Tag summit (∼100–200 bp) exhibited robust basal activity, and *SOX5* knockdown significantly increased reporter signal (*p* < 0.01) (Figure 4G). As a complementary analysis, the PCNP promoter‐proximal region showed concordant SOX5‐associated signal, reduced ATAC accessibility, and decreased PCNP transcription in SOX5‐overexpressing cells, and structural modeling further supported a plausible SOX5–PCNP promoter interface (Figure S4E,F). In addition, mutation of the predicted SOX motif within the PCNP promoter reporter weakened the response to *SOX5* perturbation, supporting the functional relevance of this promoter element in *SOX5*‐associated PCNP repression (Figure S4G). As an exploratory extension of this strategy, we summarized CUT&Tag, ATAC‐seq, and RNA‐seq changes at the gene level using an integrated regulatory‐class framework, which stratified *SOX5*‐linked genes according to *SOX5* occupancy gain, accessibility change, and transcriptional response and retained PCNP and SMARCA2 as highlighted candidate loci for downstream validation (Figure S4H–J and Table S6).

Histopathologically, *SOX5* and *PCNP* showed extensive spatial overlap in *Myc^Cd19‐Cre^* spleens, where higher *SOX5* intensity coincided with reduced *PCNP* intensity (Figure 4H,I). To extend this observation to primary human follicular lymphoma (FL) tissue, we performed multiplex immunofluorescence. Immunofluorescence of POD24 vs. non‐POD24 cases showed higher nuclear *SOX5* and lower PCNP in germinal centers (Figure S5A,B). Together, these orthogonal lines of evidence support a *SOX5–PCNP* promoter regulatory axis within the *MYC*‐driven context.

### 2.5 The *SOX5–PCNP* Axis Enforces a Proliferative Program in *MYC*‐Driven B‐Cell Lymphoma

The biological functions of *PCNP* under *SOX5* regulation have been insufficiently defined; prior reports mainly implicate ubiquitin‐dependent proteasomal pathways in cell differentiation and proliferation [26]. In system, sg*SOX5* robustly suppressed *PCNP* expression (Figure S5C), whereas *SOX5‐ko* abolished *SOX5* occupancy at the *PCNP* locus and increased *PCNP* expression (Figure 4J), coinciding with a pronounced anti‐proliferative phenotype (Figure 4K). Apoptosis tended to increase (Figure 4L), and the cell cycle showed substantial G2/M arrest (Figure 4M and Figure S5D), supporting the *SOX5–PCNP* axis as a mediator constraining lymphoma proliferation when *SOX5* is inhibited.

These data are consistent with a role for *SOX5* in supporting the proliferative state, while temporal perturbation studies will be required to distinguish initiation‐stage effects from maintenance functions (Figure 4N).

### 2.6 *PCNP* Derepression Mediates the Anti‐Proliferative Phenotype Triggered by *SOX5* Loss

**FIGURE 5:**
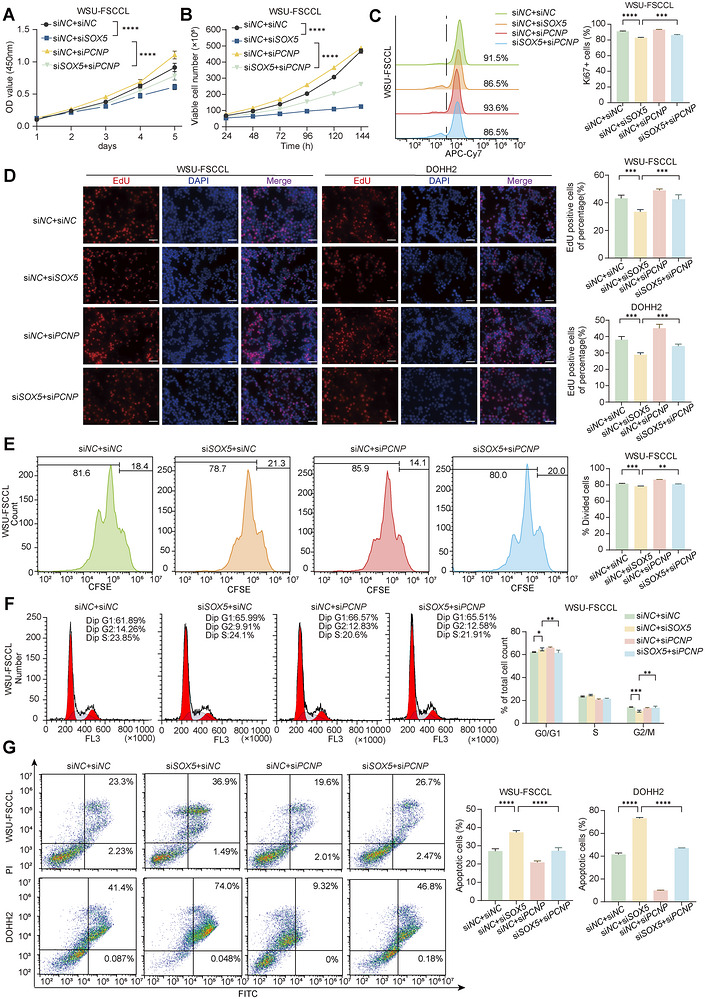
Rescue analysis positions *PCNP* downstream of *SOX5* in sustaining the proliferative state of B‐cell lymphoma cells. (A) CCK‐8 assays showing cell viability at the indicated time points following transfection. (B) Cell counting–based growth curves across the indicated time window in WSU‐FSCCL cells. (C) Flow cytometric analysis of Ki67 with quantification of Ki67‐positive fractions. (D) EdU incorporation assays with representative images and quantification of EdU^+^ cells. Scale bar, 15 µm. (E) CFSE dilution–based proliferation tracing with quantification of division profiles in WSU‐FSCCL cells. (F) Cell‐cycle distribution by flow cytometry, showing percentages of cells in G0/G1 and G2/M phases. (G) Apoptosis analysis by flow cytometry, with quantification of apoptotic fractions. Data are presented as mean ± SD from at least three independent experiments unless otherwise stated. Statistical significance was assessed by one‐way ANOVA with multiple‐comparison correction; key comparisons are indicated in the plots (ns, *p < 0.05, **p < 0.01, ***p < 0.001, ****p < 0.0001).

*SOX5* depletion derepressed *PCNP* expression, consistent with *SOX5*‐mediated promoter‐proximal repression, and this *PCNP* upregulation was abrogated upon concomitant *PCNP* silencing (Figure S6A,B). Functionally, integrated proliferation readouts collectively showed that *SOX5* knockdown markedly constrained population expansion, whereas co‐depletion of *PCNP* substantially alleviated this growth defect, restoring proliferative capacity toward control levels (Figure 5A–E and Figure S6C–F). In line with these findings, cell‐cycle profiling indicated that *SOX5* depletion shifted cells toward a less‐cycling distribution, which was partially normalized by *PCNP* co‐silencing (Figure 5F and Figure S6G), and the elevated apoptotic fraction observed upon *SOX5* knockdown was attenuated when *PCNP* was concomitantly depleted (Figure 5G). To complement the PCNP loss‐of‐function epistasis analysis, we performed reciprocal rescue experiments by restoring PCNP expression in sg*SOX5* cells. PCNP overexpression attenuated sg*SOX5*‐induced population expansion, Ki67 positivity, EdU incorporation, cell‐cycle progression, and apoptosis resistance, whereas PCNP overexpression alone produced a less proliferative phenotype relative to sg*NCs*+EV cells (Figure S7A–F). Key findings were further validated in DOHH2 cells, in which *SOX5* depletion increased PCNP expression (Figure S7A), and reduced lymphoma‐cell proliferation, supporting the broader applicability of the *SOX5*–PCNP relationship across B‐cell lymphoma contexts (Figure S7B–F). Consistently, immunoblotting showed that PCNP restoration reduced BCL2, CDK1, and MYC while increasing BAX and p21 in sg*SOX5* cells (Figure S7G,H). Together, these reciprocal epistasis and rescue data support PCNP as a functional downstream effector through which *SOX5* sustains lymphoma‐cell proliferation.

### 2.7 *SOX5* Knockdown Restores *PCNP* and Limits Lymphoma Growth Without Added Benefit From Histone Methyltransferase EZH2 Inhibition

**FIGURE 6:**
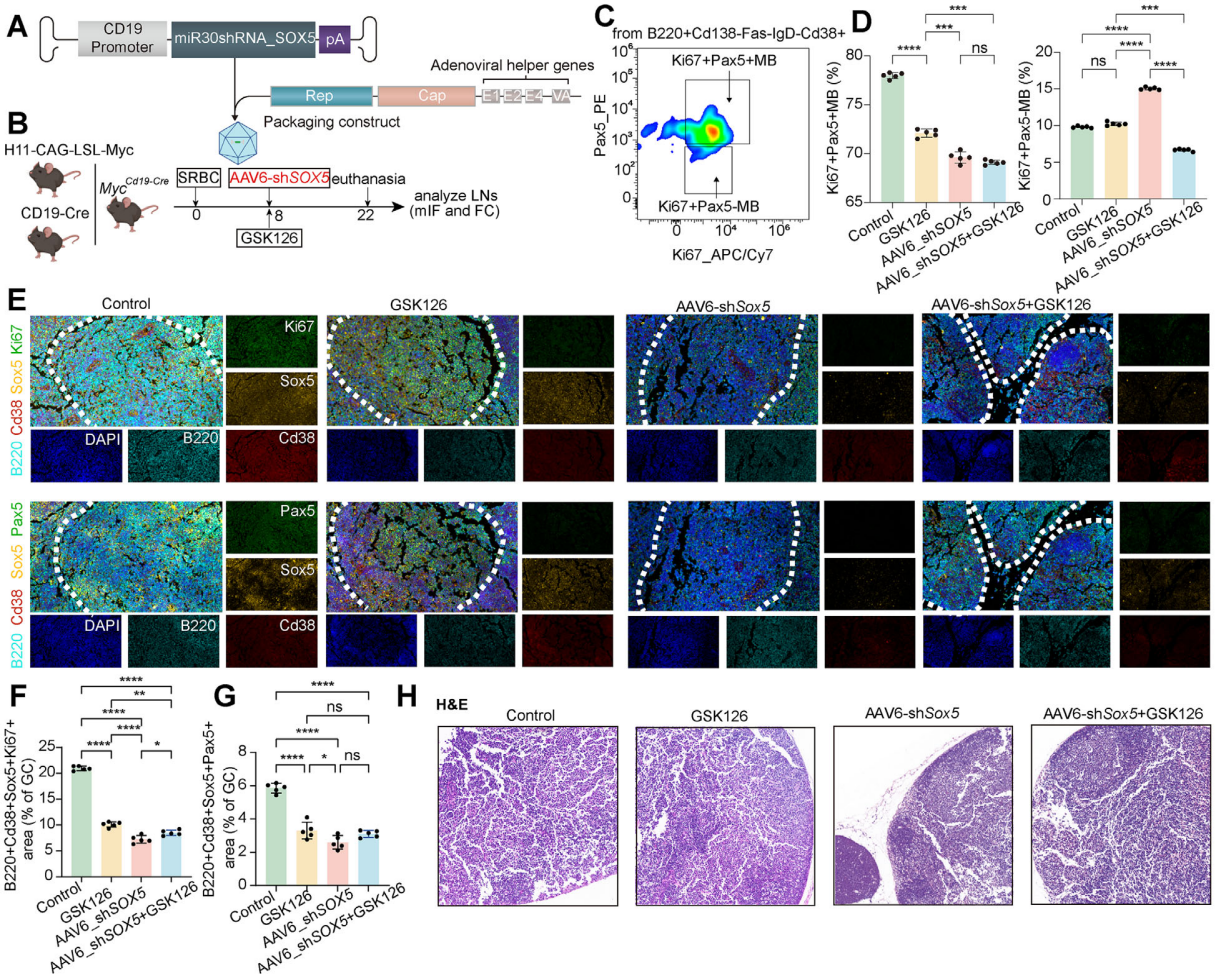
In vivo *SOX5* knockdown limits germinal‐center lymphoma proliferation; EZH2 inhibition adds no clear benefit. (A) AAV6‐sh*SOX5* vector design for B‐cell–targeted *SOX5* knockdown. (B) Experimental scheme in the *MYC*‐driven mouse model. (C) Gating strategy for Ki67‐positive memory‐B compartments among PAX5‐positive and ‐negative subclusters. (D) Quantification of the gated population shown in (C) across treatment groups: Control, EZH2 inhibitor GSK126, AAV6‐sh*SOX5*, AAV6‐sh*SOX5*+GSK126. Each dot represents one mouse. (E) Representative multiplex immunofluorescence images of germinal centers in lymph nodes for B‐cell compartments (B220, CD38, *SOX5*) with Ki67 channels (above) or PAX5 channels (below). For spatial quantification, multiple regions were averaged per mouse before statistical testing. Scale bar, 100 µm. (F,G) Spatial quantification of area fraction of B220^+^CD38^+^ *SOX5* ^+^Ki67^+^ signals and B220^+^CD38^+^ *SOX5* ^+^PAX5^+^ signals across treatment groups. (H) H&E sections are shown for morphology. Scale bar, 100 µm. One‐way ANOVA with Tukey's post hoc test was used for comparisons among three or more groups; Mann–Whitney U test or Student's *t*‐test was used for two‐group comparisons where appropriate. Significance is denoted as ns, *p < 0.05, **p < 0.01, ***p < 0.001, ****p < 0.0001.

We subsequently evaluated in vivo intervention in the *Myc^Cd19‐Cre^* model. To achieve B‐cell–specific *SOX5* knockdown, we engineered an adeno‐associated virus 6 vector [27] carrying a CD19‐promoter–driven, miR30‐based shRNA targeting *SOX5* (AAV6‐sh*SOX5*) (Figure 6A). Additional gross‐anatomical analyses supported B‐cell‐compartment delivery of AAV6‐sh*SOX5* and reduced lymph‐node burden after *SOX5*‐targeted intervention (Figure S8A). Flow‐cytometric analysis confirmed efficaciously reduced *SOX5* expression in B220+ cells after AAV6‐sh*SOX5* treatment (Figure S8B), supporting successful in vivo delivery of the AAV6‐sh*SOX5* vector. Specifically, *PCNP* expression increased within and at the margins of germinal centers in AAV6‐sh*SOX5*–treated mice relative to non‐intervention controls (Figure S8C), mirroring our cellular findings and reinforcing the centrality of the *SOX5–PCNP* regulatory relationship.

Given the clinical activity of histone methyltransferase *EZH2* inhibition in relapsed/refractory B‐cell lymphomas [28], we evaluated the in vivo effects of AAV6‐sh*SOX5*, the canonical *EZH2* inhibitor GSK126, and their combination strategy (Figure 6B). Based on our single‐cell lineage trajectory, we used *Pax5* to demarcate terminal B‐lymphoma subpopulations and defined tumorigenic memory B cells as B220^+^CD138^−^FAS^−^CD38^+^ IgD^−^ [29] (Figure 6C). Quantitatively, all three regimens significantly reduced the Ki67‐positive ratio within tumorigenic Pax5^+^ memory B cells, while the Ki67 ratio in Pax5^−^ MB cells showed a relative increase, consistent with selective suppression of malignant proliferation. Among single agents, AAV6‐sh*SOX5* produced the most pronounced suppression in most individuals; GSK126 changed in the same direction. Notably, the combination did not demonstrate a clear additive or synergistic advantage over monotherapy in this compartment (Figure 6D).

Spatial quantification of Pax5 and Ki67 within B220^+^CD38^+^ memory B cells from the same cohort corroborated these trends: both the AAV6‐sh*SOX5* and combination groups showed significant decreases in PAX5 and Ki67 signals within germinal centers (white box), whereas GSK126 exhibited a similar but weaker reduction (Figure 6E). Concordantly, spatial co‐location analysis did not reveal an advantage of the combination over AAV6‐sh*SOX5* monotherapy in reducing the proportions of PAX5^+^ and Ki67^+^ memory B cells within germinal centers (Figure 6F,G). Histopathologically, AAV6‐sh*SOX5* monotherapy and the combination partially restored follicular architecture with more defined germinal centers, with a similar but less pronounced trend under GSK126 (Figure 6H). Additional histological analyses also showed stronger architectural preservation when AAV6‐sh*SOX5* intervention was applied at the earlier disease stage, and IHC of primary human lymphoma tissues revealed a *SOX5*‐high/PCNP‐low pattern that was more evident in POD24 FL and DLBCL than in non‐POD24 FL (Figure S8D,E).

## 3 Discussion

Aberrant cell cycle progression and compromised immune surveillance constitute two core pathological hallmarks of B‐cell malignancies [30]. Against this pathophysiological backdrop, the present work identifies *SOX5* as a critical epigenetic effector sustaining MYC‐driven hyperproliferation in germinal center B cells, with its oncogenic function mediated via promoter‐proximal chromatin remodeling. Multi‐omic profiling revealed that enforced *SOX5* expression triggers de novo transcription start site‐proximal binding events, accompanied by contracted chromatin accessibility at a cohort of target regulatory loci, with *PCNP* standing out as a top functional candidate. We refrain from attributing these ATAC‐seq‐derived accessibility shifts to a single unified repressive cascade. Instead, these molecular signatures reflect a locally compacted promoter chromatin landscape, which may arise from stabilized nucleosome occupancy, diminished recruitment of transcriptional co‐activators, or cooperative assembly of repressive chromatin complexes. The enrichment of Polycomb and other repressor‐linked DNA motifs within SOX5‐gained promoter peaks lends indirect support to this working model, though direct biochemical evidence documenting physical interactions between *SOX5* and specific chromatin modifiers remains to be experimentally validated.

**FIGURE 7:**
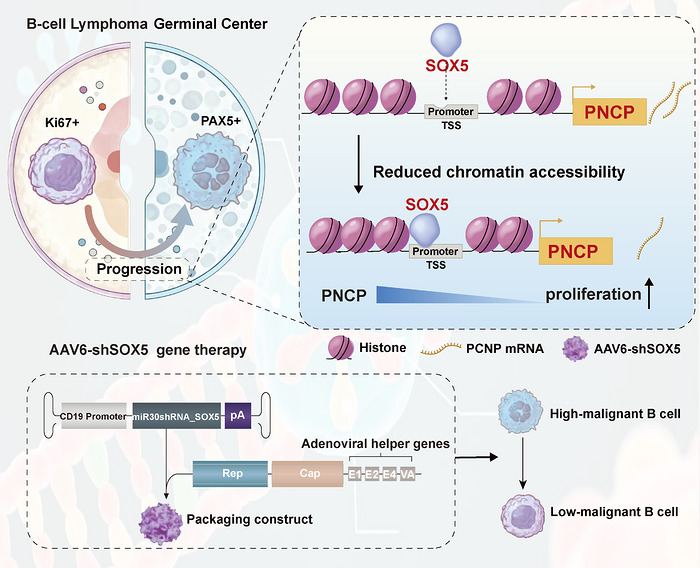
The *SOX5*–*PCNP* axis in germinal center‐derived B‐cell lymphoma and potential therapeutic strategy using AAV6‐sh*SOX5*. In *MYC*‐driven germinal‐center B cells, nuclear *SOX5* binds the *PCNP* promoter/TSS and enforces a promoter‐centric repression program characterized by de novo proximal occupancy with contraction of promoter accessibility—thereby reducing *PCNP* transcription and sustaining a Ki‐67^+^ PAX5^+^ malignant memory‐B state and GC expansion. B‐cell–targeted *SOX5* knockdown via AAV6‐sh*SOX5* relieves this repression. The *SOX5*–*PCNP* axis is proposed as a mechanistic node for therapeutic exploration.

A core mechanistic takeaway from our multi‐layered functional assays is that *SOX5* mediates transcriptional silencing of *PCNP* through direct promoter occupancy. While prior reports have implicated *PCNP* as a modulator of apoptotic signaling across multiple tumor types [31], its biological relevance and downstream signaling circuitry within B‐cell lymphoma have remained largely uncharacterized. Our orthogonal loss‐of‐function, overexpression and epistasis rescue experiments position *PCNP* as a necessary downstream effector of *SOX5*, demonstrating that modulating *PCNP* abundance reverses the proliferative and apoptotic phenotypes elicited by *SOX5* perturbation. This genetic epistasis bridges SOX5‐dependent chromatin remodeling at gene promoters to the control of malignant B‐cell expansion, establishing a linear regulatory cascade wherein *SOX5* sustains unchecked proliferation at least partially through transcriptional suppression of *PCNP*. As illustrated in Figure 7, the *SOX5*–*PCNP* axis thus provides a direct mechanistic link between epigenetic promoter regulation and cellular fitness in MYC‐transformed B cells.

The therapeutic landscape for relapsed/refractory B‐cell lymphoma has undergone transformative expansion beyond traditional chemoimmunotherapy, most notably through the clinical deployment of CD19‐targeted CAR‐T products and CD20×CD3 bispecific T‐cell engagers [4, 32, 33, 34]. Distinct from these cell‐surface‐directed modalities, the SOX5–PCNP axis represents an intracellular epigenetic vulnerability that could be exploited for alternative therapeutic intervention. *PCNP* encodes a nuclear protein bearing a PEST degradation motif, whose biological outputs are highly context‐dependent across human malignancies; loss of *PCNP* expression has consistently correlated with aggressive clinical phenotypes in multiple solid and hematologic neoplasms [35]. Existing mechanistic work has tied *PCNP* activity to autophagic turnover and apoptotic signaling cascades governed by Wnt/β‐catenin and MAPK kinase pathways [36]. Consistent with these published observations, our multi‐omic datasets demonstrate that *SOX5* accumulation at proximal promoter regions coincides with chromatin compaction and transcriptional repression of a suite of genes governing apoptosis, cell cycle progression and cellular turnover [25]. These results integrate *SOX5* into a broader epigenetic network governing the growth homeostasis of malignant B cells [37], yet the full spectrum of SOX5‐bound cis‐regulatory elements and target genes across diverse lymphoma genetic subtypes awaits comprehensive profiling.

Contemporary advances in viral gene delivery have unlocked new opportunities to therapeutically target intracellular transcription factors and epigenetic regulators in intact preclinical models [38], Here, we leveraged a CD19 promoter‐driven, miR30‐shSOX5 expression cassette packaged within an AAV6 vector to achieve selective silencing of *SOX5* specifically in B‐lineage cells of our Myc^Cd19‐Cre^ transgenic lymphoma model. Systemic AAV6‐shSOX5 administration effectively reduced the pool of Ki67‐expressing proliferative malignant B cells and partially restored the disrupted follicular tissue architecture, exerting more potent anti‐tumor effects than single‐agent *EZH2* inhibition under our standardized experimental regimens [39]. These preclinical observations validate *SOX5* as a pharmacologically tractable therapeutic target in this MYC‐driven lymphoma system. Nevertheless, several translational hurdles limit immediate clinical translation of AAV6‐based *SOX5* knockdown. High baseline seroprevalence of neutralizing antibodies against AAV serotypes including AAV1 and AAV6 blunts cellular transduction efficiency in human patient populations; future translational work will therefore require refined strategies such as patient serostratification, transient immunosuppressive conditioning, capsid protein engineering, or the development of non‐viral delivery platforms to circumvent this limitation [40].

Several limitations should be noted. First, the in vivo evidence is derived from a single MYC‐driven lymphoma model and a focused set of therapeutic endpoints; validation across additional genetic backgrounds, treatment‐resistant settings, and primary patient cohorts will be required to define the broader applicability of the SOX5–PCNP axis. Second, although our data link SOX5‐associated promoter occupancy to reduced accessibility and *PCNP* repression, the chromatin cofactors that cooperate with *SOX5* at promoter‐proximal regions remain to be identified biochemically. Third, deeper profiling of immune and stromal compartments may clarify how SOX5‐directed intervention reshapes the lymphoma microenvironment. In summary, our findings define a SOX5‐associated promoter‐regulatory program that represses *PCNP* and supports proliferative‐state maintenance in GC‐derived lymphoma, and they provide in vivo evidence that *SOX5* knockdown can constrain this program, nominating the SOX5–PCNP axis for further therapeutic investigation.

## 4 Materials and Methods

### 4.1 Mice and In Vivo Interventions

*Myc^Cd19‐Cre^* mice (strain number CM‐241142) were obtained from the Southern Model Organism Center (Shanghai) and generated according to the supplier's published design. The H11‐CAG‐LSL‐Myc allele was generated on a C57BL/6J background by CRISPR/Cas9‐mediated homologous recombination at the Hipp11 locus and contains a CAG‐loxP‐PGK‐Neo‐pA‐loxP‐Myc‐WPRE‐pA cassette. Before Cre recombination, the loxP‐flanked PGK‐Neo‐pA stop cassette blocks Myc expression; Cd19‐Cre‐mediated excision activates the CAG‐driven Myc allele in the B‐cell lineage. Wild‐type controls were age‐ and sex‐matched C57BL/6J mice maintained under the same SPF housing and SRBC‐immunization conditions as the MycCd19‐Cre mice. This MYC‐driven model has been previously characterized by lymphoid‐organ enlargement, germinal‐center expansion, histopathological disruption, and lymphoma‐associated B‐cell expansion under immunization conditions. Tumor‐bearing or lymphoma‐associated status was defined by lymphoid‐organ enlargement together with histopathological and immunophenotypic evidence of B‐cell expansion, including disrupted lymphoid architecture on H&E sections, expansion of B220/PAX5‐positive B‐cell regions, and increased Ki67‐positive proliferative activity. The phenotype of this model was confirmed in our previous study [12], and the corresponding single‐cell sequencing data are publicly available at Zenodo (15301238). Mice were housed in the SPF‐grade animal facility at the Second Affiliated Hospital of Harbin Medical University. All animal experiments were approved by the Institutional Animal Care and Use Committees of the Southern Model Organism Center and Harbin Medical University (approval number HMUIRB2025004; license number SCXK[Hu]2019‐0002). Mice were randomized into groups prior to experiments and maintained in an SPF facility with a 12‐h light/dark cycle. To induce germinal centers (GCs), 8–12‐week‐old mice received an intraperitoneal injection of 2% sheep red blood cells (SRBC; BERSEE) on Day 0. Drug interventions were administered on day 8: cohorts included control, AAV6‐sh*SOX5*, GSK126, and combination groups. AAV6‐sh*SOX5* was delivered as a single tail‐vein injection at 2.5 × 10^1^ ^1^ genome copies (GC) per mouse (≈8.33 × 10^1^ ^2^ GC/kg) in 0.9% saline. GSK126 was given intraperitoneally at 30 mg/kg, twice weekly for two consecutive weeks (four total doses), formulated in 20% (w/v) sulfobutylether‐β‐cyclodextrin (SBE‐β‐CD) in 0.9% saline. The combination group received both on the same schedules; vehicle volumes were matched to route and excipient. Spleens and superficial lymph nodes were harvested on day 21 for analysis.

### 4.2 Cell Culture and Gene Perturbations (siRNA, CRISPRa)

Human follicular lymphoma cell lines WSU‐FSCCL (DSMZ) and DOHH2 (Procell) were authenticated by STR profiling and confirmed mycoplasma‐negative. Cells were cultured in RPMI‐1640 medium supplemented with 10% FBS at 37°C in a 5% CO_2_ humidified incubator.

#### 4.2.1 siRNA Transfection

To knock down *SOX5* in WSU‐FSCCL cells, siRNA transfection was performed using GP‐transfect‐Mate (GenePharma) according to the manufacturer's instructions. si‐*SOX5* or si‐NC was diluted in serum‐free medium with transfection reagent, incubated at room temperature for 15–20 min to form complexes, and added to cells at 50%–60% confluence in six‐well plates. Cells were harvested after 48 h, and knockdown efficiency was verified by RT‐qPCR.

#### 4.2.2 CRISPRa‐Mediated Activation

The SAM‐CRISPRa system was used for lentiviral co‐infection to activate endogenous *SOX5* in WSU‐FSCCL cells. Experimental groups were infected with lenti sgRNA (MS2) zeo carrying *SOX5* sgRNA, MS2‐P65‐HSF1_Hygro, and dCas‐VP64_Blast; control groups used nonsense sgRNA vectors with identical auxiliary components. Medium was replaced after 24 h, followed by selection with Zeocin, Hygromycin, and Blasticidin for 5–7 days until negative controls died. Positive cells were recovered for 1–2 days, and *SOX5* expression was assessed by RT‐qPCR. All lentiviral vectors were from Vigene.

### 4.3 Suppression‐Epistasis Analysis of the *SOX5*–*PCNP* Axis

For suppression‐epistasis analysis, cells were transfected with siRNAs in four conditions: siNC+siNC, si*SOX5*+siNC, siNC+siPCNP, and si*SOX5*+siPCNP. Unless otherwise indicated, the siRNA oligonucleotides, transfection reagent, and final siRNA concentrations were identical to those described in the “siRNA Transfection” section above. Knockdown efficiency was assessed by RT‐qPCR at 48 h post‐transfection. For functional readouts, CCK‐8 assays were performed at 0, 24, 48, 72, and 96 h post‐transfection, and cell counting–based growth curves were recorded at 24, 48, 72, and 96 h. EdU incorporation and Ki67 staining were assessed at 48 h post‐transfection. For CFSE‐based proliferation tracing, cells were labeled with CFSE immediately after transfection and division profiles were analyzed at 72 h. Cell‐cycle distribution and apoptosis were quantified by flow cytometry at 48 h post‐transfection. Each experiment was repeated independently at least three times unless otherwise stated.

### 4.4 Histology, IHC, and Multiplex IF

Spleens and lymph nodes were fixed in 4% paraformaldehyde for 24 h, dehydrated in graded ethanol, paraffin‐embedded, and sectioned at 5 µm. H&E staining was used to examine tissue morphology. For IHC, sections were deparaffinized, rehydrated, antigen‐retrieved in citrate buffer (pH 6.0), blocked with hydrogen peroxide and 3% BSA, incubated with primary antibodies at 4°C overnight, washed with PBST, incubated with HRP‐conjugated secondary antibodies, developed with DAB, and counterstained with hematoxylin.

For multiplex immunofluorescence, cell smears and tissue sections were deparaffinized, rehydrated, permeabilized, antigen‐retrieved, and endogenous peroxidase‐blocked. After blocking with 3% BSA or normal serum, sections were incubated with primary antibodies at 4°C overnight, followed by HRP‐conjugated secondary antibodies and TSA signal amplification; antibodies were stripped with elution buffer after TBST washes, and the process repeated for the second antigen. The final antigen was detected with fluorescent secondary antibodies (no TSA). Nuclei were stained with DAPI, mounted with anti‐fade reagent, and imaged. Antibody details are provided in the Key Resources Table (Table S1).

### 4.5 Sequencing and Computational Analysis (CUT&Tag, ATAC‐seq, RNA‐Seq)

#### 4.5.1 CUT&Tag

The NovoNGS CUT&Tag 4.0 High‐Sensitivity Kit (Illumina, N259‐YH01) was used according to the manufacturer's instructions. 1 × 10^5^ cells were fixed to ConA beads, incubated with primary antibodies at 4°C overnight (anti‐*SOX5* antibody, CST‐25792T) and secondary antibodies (PTM‐6252) at room temperature for 60 min. pA‐Tn5 was added for 1 h incubation and tagmentation at 37°C. DNA was purified, PCR‐amplified to construct libraries, quality‐checked, and sequenced on Illumina NovaSeq 6000 (PE150). Raw reads were processed with Cutadapt to remove adapter sequences and reads with length <30 bp, quality score <30, or an N‐base proportion >5%, and the cleaned reads were aligned to hg38 or mm10 (sample‐dependent) using Bowtie2. Peaks were called with MACS2 (q < 0.05), motifs analyzed with HOMER (v4.11.1, *SOX5*‐enriched homerResults of de novo unknown 41 and knownResults of public 176), and annotated with ChIPseeker. Differentially accessible regions were merged with bedtools and analyzed by DESeq2 (|log_2_FC| > 1, *p* < 0.05). GO and KEGG enrichment analyses of genes near differential peaks were performed using clusterProfiler. Antibody details are provided in the Key Resources Table.

#### 4.5.2 Bulk ATAC‐Seq

*SOX5*‐overexpressing WSU‐FSCCL cells were collected after 48 h (viability ≥80%). 5 × 10^4^ cells were lysed for nuclei isolation; tissue samples were homogenized, filtered, and processed similarly. Open chromatin was tagmented with Tn5, and libraries constructed using TruePrep DNA Library Prep Kit V2 (TD501) for PE150 sequencing on Illumina X Plus. Data were trimmed with Trim Galore, aligned to hg38 with Bowtie2, deduplicated with SAMtools. Peaks were called with MACS2 (parameters referenced from CUT&Tag). Signal tracks and heatmaps were generated with deepTools and annotated with ChIPseeker. Differential accessibility was analyzed with DESeq2 (|log_2_FC|>1, FDR<0.05), and GO/KEGG terms enrichment analyses of genes near differential peaks were performed using clusterProfiler.

#### 4.5.3 Bulk RNA‐Seq

Total RNA from *SOX5*‐overexpressing or knockdown WSU‐FSCCL cells (RIN>7) was extracted. mRNA was enriched with oligo(dT) beads, fragmented, reverse‐transcribed to cDNA, and libraries constructed. Quality‐checked libraries were sequenced on Illumina NovaSeq 6000 (PE150). Data were processed with fastp/Trim Galore, aligned to hg38/mm10 (sample‐dependent) using HISAT2/STAR. Reads were counted with featureCounts/HTSeq and normalized to FPKM. Differential expression was analyzed with DESeq2 (adjusted *p* < 0.05, |log_2_FC|>0.5), followed by GO/KEGG enrichment with clusterProfiler/Metascape (Benjamini–Hochberg correction).

### 4.6 Flow Cytometry and Functional Assays

For mouse samples, single‐cell suspensions were prepared from superficial lymph nodes of the GSK126, AAV6‐sh*SOX5*, combination, and control groups, treated with red blood cell lysis buffer (TBD Science), and resuspended in cell staining buffer (BioLegend). Fc receptors were blocked with 2.4G2 (BioXcell), followed by staining with fluorophore‐conjugated antibodies. Doublets were excluded using FSC‐W/FSC‐H gating. Samples were acquired on a BD FACSCanto II or BD LSRFortessa and analyzed with FlowJo software (BD Biosciences).

For cell‐line assays, EdU incorporation was performed. Briefly, sg*SOX5*/si*SOX5* and control WSU‐FSCCL and DOHH2 cells were seeded in 96‐well plates and incubated with EdU for 2 h, followed by fixation, permeabilization, Click‐iT reaction, and DAPI counterstaining. Images were acquired on an EVOS FL Auto (Thermo Fisher Scientific), and EdU‐positive ratios were quantified. For epistasis experiments, EdU incorporation was measured at 48 h post‐transfection across four siRNA conditions (siNC+siNC, si*SOX5*+siNC, siNC+siPCNP, and si*SOX5*+siPCNP).

For Ki67 analysis under the same epistasis design, cells were stained with anti‐Ki67 antibody and quantified by flow cytometry at 48 h post‐transfection. For CFSE‐based proliferation tracing (CFSE Cell Division Tracker Kit, Elabscience Biotechnology Co., Ltd., Cat. No. E‐CK‐A345), cells were labeled with CFSE immediately after transfection and division profiles were analyzed at 72 h.

Apoptosis was assessed by Annexin V/PI staining (FITC Annexin V Apoptosis Detection Kit I, BD Pharmingen) and analyzed by flow cytometry at 48 h post‐transfection under the epistasis design. For cell‐cycle analysis, cells were fixed in cold 95% ethanol, stained with PI/RNase A for 30 min at 37°C in the dark, filtered, and analyzed by flow cytometry to determine the G0/G1, S, and G2/M distributions at 48 h post‐transfection.

### 4.7 Statistical Analysis

All experiments were performed in at least three independent biological replicates. Normally distributed data are presented as mean ± SD; non‐normal data as median (IQR). Analyses were conducted using GraphPad Prism 9.5.0 and SPSS 29.0. Two‐group comparisons used two‐sided Student's *t*‐test or Mann–Whitney U test; paired data used Wilcoxon signed‐rank test; multiple groups used one‐way ANOVA with Tukey's post hoc test where appropriate. Unless stated otherwise, tests were two‐sided with *p* < 0.05 considered significant (*p < 0.05, **p < 0.01, ***p < 0.001, ****p < 0.0001). Exact sample sizes, statistical methods, and *p*‐values are indicated in figures or legends.

## Author Contributions

Conceptualization: Y.W.D, Q.Y.Z., J.L.M.; Methodology: Y.Y.M., Z.Y.J., X.Q.M.; Investigation: R.L.W.; Visualization: Y.Y.M.; Supervision: Y.W.D.; Writing – original draft: Y.Y.M, Z.Y.J. Writing – review & editing: Y.W.D., Q.Y.Z., J.L.M. All authors have read and approved the final version of the manuscript.

## Funding

This work was supported by the Youth Project of the National Natural Science Foundation of China No.82303299 (YWD) and the Heilongjiang Provincial Natural Science Outstanding Youth Project No. YQ2023H021 (JLM), Haiyan Foundation Outstanding Young Scholar Program No. JJYQ2024‐01 (YWD), Key Research and Development Program Project of Heilongjiang Province No. 2023ZX06C08 (QYZ) and the New Era Longjiang Excellent Doctoral Dissertation Project No. LJYXL2022‐65 (YWD).

## Ethics Approval and Consent to Participate

The experimental procedures were performed in strict accordance with the institutional guidelines delineated by Harbin Medical University, the Guide for the Care and Use of Laboratory Animals, and standards established by the Association for Assessment and Accreditation of Laboratory Animal Care International. Southern Model Organism Center (Shanghai), the Institutional Animal Care and Use Committee of Harbin Medical University, and Harbin Veterinary Research Institute, Chinese Academy of Agricultural Sciences, vetted all procedures and duly approved the entirety of the study involving mice under institutional Review Boards #HMUIRB2025004 and protocols #SCXK(shanghai)2019‐0002. All study procedures for human samples were approved by the Institutional Review Boards (IRBs) of Harbin Medical University Cancer Hospital (approval no. KY2023‐79). This research has been registered for clinical study in the Medical Research Registration Information System. The registration number is: MR‐23‐25‐023947.

## Conflicts of Interest

The authors declare no conflicts of interest.

## Supporting information




**Supporting file 1**: advs76656‐sup‐0001‐SuppMat.docx.

**Supporting file 2**: advs76656‐sup‐0002‐FigureS1–S8.zip.

**Supporting file 3**: advs76656‐sup‐0003‐TableS1–S6.zip.

## Data Availability

The data that support the findings of this study are openly available in Zenodo at https://zenodo.org, reference number 10.5281/zenodo.17383655;17385283;17389142;15301238.
