# Supplementary material for: SOX5 Orchestrates Malignant Evolution via Promoter‐Centric Chromatin Remodeling in MYC‐Driven B‐Cell Lymphoma
Source: Adv Sci (Weinh). 2026 Jul 17;13(57):e76656. doi: 10.1002/advs.76656 (PMC13379260; doi:10.1002/advs.76656)
Supplement: Supplementary file 1 [file ADVS-13-e76656-s003.docx]

**Supplementary informations**

**Figure S1. Genetic, histopathological, and B-cell-compartment validation of the *Myc^Cd19-Cre^* model.** (A) Genotyping PCR of the transgenic founders. Representative gel images with ladder sizes (bp) are displayed. (B) Genotyping validation of the H11-CAG-LSL-Myc allele. Representative PCR electrophoresis showing 5′ and 3′ homology-arm amplification in F1 mice, with sequencing-confirmed positive insertion, and representative F2/F3 genotyping confirming transmission of the intended allele. (C) Anatomical presentation of the inguinal lymph nodes (The yellow arrow indicates the lymph node position), and measurement of spleen and inguinal lymph node size in mice. (D) Representative H&E staining of lymphoid tissues from wild-type and *Myc^Cd19-Cre^* mice. *Myc^Cd19-Cre^* tissues showed lymphoma-associated architectural alteration with abnormal lymphoid expansion, mixed cellularity, atypical lymphoid cells, and focal degenerative/necrotic changes. Scale bars, 100 μm (top row), 50 μm (middle row), and 20 μm (bottom row). (E) Flow-cytometric sorting strategy for isolating lymph-node B220⁺ B cells from wild-type and *Myc^Cd19-Cre^* mice. (F) qRT-PCR analysis of *Myc* mRNA expression in flow-sorted lymph-node B220⁺ B cells. (G) Immunoblot analysis of Myc protein expression in flow-sorted lymph-node B220⁺ B cells. (H) Multiplex immunofluorescence showing Myc and B220 localization in lymphoid tissues. B220 was detected as green membrane staining, and B220-positive B cells in *Myc^Cd19-Cre^* tissues displayed high intranuclear Myc expression signals (yellow). DAPI marks nuclei. Scale bars, 20 μm.

**Figure S2.** **Single-cell characterization, reference mapping, and outcome relevance of the SOX5-enriched proliferative B-cell state.** (A) UMAP visualization of single-cell transcriptomes, showing B-cell subclusters and the discrete cluster 12 (Mki67⁺ memory-B) (yellow box). (B) Violin plots showing normalized expression for Mki67 and *SOX5* across selected clusters annotated by canonical markers: 0 (Hdac9⁺ GC-B), 1 (Cenpa⁺ GC-B), 8 (Ifit3⁺ GC-B), 9 (Pax5⁺ memory-B), and 12 (Mki67⁺ memory-B). (C) Kaplan–Meier survival analysis of mouse survival. (D) Gene set enrichment analysis (GSEA) related to lineage and differentiation programs. Running Enrichment Score plots with Ranked List Metric for the indicated terms. “Skeletal system morphogenesis” shows no significant enrichment (p=0.942, p.adjust=0.942), whereas “stem cell differentiation” showed a trend toward enrichment (p=0.0281, p.adjust=0.0561), as displayed on the plots. (E) Kaplan–Meier survival analysis of human diffuse large B-cell lymphoma stratified by *SOX5* expression in TCGA. (F) Scatter plot of *SOX5* expression across B-cell subclusters. (G) GO biological process enrichment associated with the *SOX5*-high proliferative state. Bar plot of top terms (Count on x-axis; color indicates p.adjust). (H) UMAP of the query dataset after Seurat anchor-based label transfer from the wild-type reference (I). (J) Cluster-wise composition of transferred labels. Cluster 12 was enriched for Early_Centroblast, Centroblast, Transitioning_CB_CC, Centrocyte, and Prememory labels, supporting its placement along a GC-to-prememory B-cell transition continuum.

**Figure S3. Transcriptome shifts, motif landscape, and promoter-proximal accessibility changes accompanying *SOX5* activation.** (A) Principal component analysis of bulk RNA-seq (sg*SOX5* vs NC). (B) Volcano plot of differential expression (DESeq2) of RNA-seq. panel thresholds: p<0.05 and |log2FC|>0. (C) Heatmap of gene expression highlighting G1/S-specific transcriptional programs under sg*SOX5* (log2(RPKM+1) scale shown). (D) Summary of increased *SOX5* occupancy signals by CUT&Tag in sg*SOX5* versus NC cells. (E) Pie chart showing background peak distribution in the control group. (F) Violin plot showing overlap between sg*SOX5*-gained signals and H3K4me3 promoter marks (ENCODE; enrichment P=0.05 as indicated) and sg*SOX5*-gained signals. (G) Representative enriched motif associated with sg*SOX5*-gained peaks. The top enriched motif is Trl(Zf)/S2–GAGA factor (rank 1; score 0.78; P=1e−13, 43.75% of targets). (H) SOX5/SOX-family motif comparison in retained sgSOX5-gained peaks. *SOX5*-associated differential peaks showed enrichment of GAGA-related motifs, including Trl-GAGA factor, and a Polycomb-associated NANOG motif in selected gained-peak sets. (I) HOMER motif analysis of the retained sgSOX5-gained peak set identified a representative de novo motif, 1-GGTATATTGTTG, whose sequence and reverse-complement orientation showed similarity to reported SOX5/SOX-family position-weight matrices, including PB0072.1 Sox5_1/JASPAR and Sox5/MA0087.1/JASPAR. The reverse-complement sequence also showed similarity to the SOX5 reference motifs provided in HumanTF/JASPAR-related resources. (J) Box plots summarizing ATAC-seq signals at candidate loci (sg*SOX5* vs control). (K) TSS-centered ATAC-seq average profiles (±3 kb) showing altered promoter-proximal accessibility under sg*SOX5* relative to NC. (L) ATAC-seq differential analysis (DESeq2/ DiffBind): volcano plot showing log2 fold change versus −log10(FDR). (M) KEGG enrichment for sg*SOX5*-regulated sets from ATAC-DEseq (categories and –log10(p) values shown).

**Figure S4. Orthogonal support for the *SOX5*–*PCNP* axis: locus-level views, cross-modality concordance, and functional validation.** (A) Venn diagram counts summarizing the common genes/signals among ATAC-seq, CUT&Tag, and RNA-seq for the ALL, UP, and DOWN gene sets (counts as displayed in the panel). (B) Correlation analysis showing a significant association (P=0.038) between the *SOX5* signature and the *PCNP* or SMARCA2 signatures. (C) IGV tracks sg*SOX5*-gained signals across the *SOX5* locus (coordinate scale shown; ~24,460–24,600 kb). (D) Heatmap and signal distribution plot showing peak-centered aggregate profiles (±5 kb around the indicated start–end coordinates) for PCNP under sg*SOX5* versus control. (E) Aggregate ATAC-seq signal profiles centered on promoter regions with or without *SOX5*-gained occupancy. Control and *SOX5*-activated/OE cells are shown across ±2 kb from the center of the indicated promoter regions. (F) Boxplot showing ATAC-seq log2 fold-change values between *SOX5*-activated/OE and control cells at *SOX5*-gained and non-gained promoter regions. *SOX5*-gained promoters showed a greater reduction in ATAC accessibility than non-gained promoters, with median log2FC values of −0.443 and −0.014, respectively. Each dot represents one promoter region. (G) Schematic of the *PCNP* promoter (~2 kb) and reporter design. A promoter fragment centered on the CUT&Tag summit was cloned into pGL4.10 to assess transcriptional output under *SOX5* perturbation. Luciferase reporter assay for the *PCNP* promoter under sg*SOX5* vs sg*NCs*; *SOX5* knockin significantly decreases reporter activity. (H) Barplot summarizing the number of genes assigned to integrated regulatory classes based on CUT&Tag, ATAC-seq, and RNA-seq patterns. (I) UpSet plot showing intersections among CUT&Tag gain, ATAC accessibility changes, and RNA expression changes. (J) Quadrant analysis integrating ATAC-seq and RNA-seq changes for SOX5-associated genes. PCNP and SMARCA2 are highlighted as candidate SOX5-associated genes prioritized for downstream validation.

**Figure S5**. **Orthogonal validation of the SOX5–PCNP regulatory relationship in human FL tissues and lymphoma cells.** (A-B) Multiplex immunofluorescence for *SOX5/PCNP/DAPI* in human follicular lymphoma with or without POD24 and quantification of nuclear *SOX5* and *PCNP* intensities, showing higher *SOX5* and reduced *PCNP* in POD24 cases. (C) Validation of *PCNP* repression in sg*SOX5* cells. Left: RT-qPCR bar plot of *PCNP* mRNA (fold) in NC vs sg*SOX5*. Right: immunoblot of *PCNP* with GAPDH in NC vs sg*SOX5*. (D) Cell-cycle effects of *SOX5*-CRISPRko.

**Figure S6. Suppression epistasis places *PCNP* downstream of *SOX5* loss in growth control.** (A-B) RT-qPCR analysis of *SOX5* and *PCNP* mRNA levels to verify knockdown efficiency and to assess *PCNP* regulation upon *SOX5* silencing. (C–D) Proliferation analyses in DOHH2 cells showing altered population expansion upon *SOX5* knockdown and its modulation by concomitant *PCNP* depletion, as assessed by CCK-8 assays and cell counting–based growth curves. (E) Flow cytometric analysis of Ki67 illustrating changes in the proportion of cycling cells under the indicated conditions. (F) CFSE-based division tracing depicting differences in cell division progression following *SOX5* knockdown and *PCNP* co-depletion. (G) Flow-cytometric cell-cycle analysis showing shifts in G0/G1 and G2/M phases across the indicated conditions. Quantifications are presented as mean ± SD with two-sided tests; significance is annotated in panels as ns, *p < 0.05, **p < 0.01, ***p < 0.001, ****p < 0.0001.

**Figure S7. *PCNP* restoration attenuates *SOX5*-induced proliferative phenotypes in B-cell lymphoma cells.** (A) qRT-PCR validation of SOX5 activation and PCNP overexpression in the indicated experimental groups. (B) Cell counting–based growth curves showing viable cell expansion over time in WSU-FSCCL and DOHH2 cells. (C) Flow-cytometric analysis of Ki67-positive fractions across the four conditions. (D) EdU incorporation assays with representative images and quantification of EdU-positive cells. Scale bar, 15 μm. (E) PI-based cell-cycle analysis showing the distribution of cells in G0/G1 and G2/M phases. (F) Annexin V/PI flow-cytometric analysis of apoptotic cells. (G–H) Immunoblot analysis of proliferation-, cell-cycle-, and apoptosis-associated markers, including *MYC, CDK1, p21, BCL2*, and *BAX,* in WSU-FSCCL (G) and DOHH2 (H) cells. ACTB was used as the loading control. Experimental groups were sg*NCs*+EV, sg*SOX5*+EV, sg*NCs*+*PCNP*-OE, and sg*SOX5*+*PCNP*-OE. Data are presented as mean ± SD from at least three independent biological replicates unless otherwise stated. Statistical significance was assessed by two-way ANOVA for time-course growth curves and by one-way ANOVA with multiple-comparison correction for endpoint assays; significance is denoted as ns, *p < 0.05, **p < 0.01, ***p < 0.001, ****p < 0.0001.

**Figure S8. Additional in vivo and human tissue validation of SOX5-targeted intervention and the SOX5–PCNP axis.** (A) Gross images of superficial lymph nodes and spleens from Control, GSK126, AAV6-shSox5, and AAV6-shSox5+GSK126 groups, with quantification of lymph node weight and spleen-to-body weight ratio. (B) Flow-cytometric analysis and quantification of B220⁺Sox5⁺ cells in lymphoma-bearing mice from the indicated treatment groups. (C) Multiplex immunofluorescence in MycCd19-Cre spleens showing representative DAPI/PCNP merged images and quantification of PCNP-positive area within germinal-center regions in Control and AAV6-shSox5-treated mice. Scale bar, 100 μm. (D) H&E staining of spleen sections from early- and late-stage AAV6-shCtrl or AAV6-shSOX5 intervention groups, with magnified views of the boxed regions. Scale bar, 100 μm. (E) IHC analysis of SOX5 and PCNP expression in primary lymphoma tissues from FL non-POD24, FL POD24, and DLBCL cases, with quantification of SOX5-positive and PCNP-positive cells. Data are presented as mean ± SD. Statistical significance was assessed using two-sided tests or one-way ANOVA with multiple-comparison correction as appropriate; ns, not significant; *p < 0.05, **p < 0.01, ***p < 0.001, ****p < 0.0001.

**Table S1. Gene ranking of cluster 12 from dataset Zenodo. 15301238.**

**Table S2. DEGs of CUT&Tag-seq in sg*SOX5* vs control.**

**Table S3. Cluster 1 gene panel related to figure 3Q (top 30).**

**Table S4. Cluster 2 gene panel related to figure 3Q (top 30).**

**Table S5. Cluster 3 gene panel related to figure 3Q.**

**Table S6. Triple Omics Integration.**
