# Supplementary material for: SOX5 Orchestrates Malignant Evolution via Promoter‐Centric Chromatin Remodeling in MYC‐Driven B‐Cell Lymphoma
Source: Adv Sci (Weinh). 2026 Jul 17;13(57):e76656. doi: 10.1002/advs.76656 (PMC13379260; doi:10.1002/advs.76656)
Supplement: Supplementary file 3 [file ADVS-13-e76656-s002.zip › Table S5.docx]

**Table S5. Cluster 3 gene panel related to figure 3Q.**

|  | log2(fpkm+1) | | | |  |
| --- | --- | --- | --- | --- | --- |
| gene | sgSOX5-1 | sgSOX5-2 | NC-1 | NC-2 | gene_biotype |
| IGLL5 | -1.527525372 | -2.064978904 | 1.80758389694554 | 1.78492037926389 | protein_coding |
| TPSP2 | -1.316142417 | -1.710492762 | 1.6264965707825 | 1.40013860761858 | protein_coding |
| TRBC2 | -0.463961969 | -1.925272355 | 1.21222142512586 | 1.17701289903877 | protein_coding |
| IGLC2 | -0.90645201 | -1.632543262 | 1.19640905963077 | 1.34258621171049 | protein_coding |
| MIR548AR | -1.900784913 | -1.900784913 | 2.5921084684714 | 1.20946135774774 | protein_coding |
| IGHJ3P | -0.600621522 | -2.810578618 | 2.22886920159892 | 1.18233093809355 | protein_coding |
| FAM27B | -0.129545828 | -1.984100483 | 1.5426504702537 | 0.570995840626114 | protein_coding |
| Y_RNA | -1.447514181 | -1.447514181 | 1.37312219115113 | 1.52190617181649 | protein_coding |
