# Supplementary material for: SOX5 Orchestrates Malignant Evolution via Promoter‐Centric Chromatin Remodeling in MYC‐Driven B‐Cell Lymphoma
Source: Adv Sci (Weinh). 2026 Jul 17:e76656. Online ahead of print. doi: 10.1002/advs.76656 (PMC13379260; doi:10.1002/advs.76656)
Supplement: Supplementary file 2 — Supporting file 2: advs76656‐sup‐0002‐FigureS1–S8.zip. [file ADVS-9999-e76656-s003.zip › Figure S1.pdf]

Figure S1

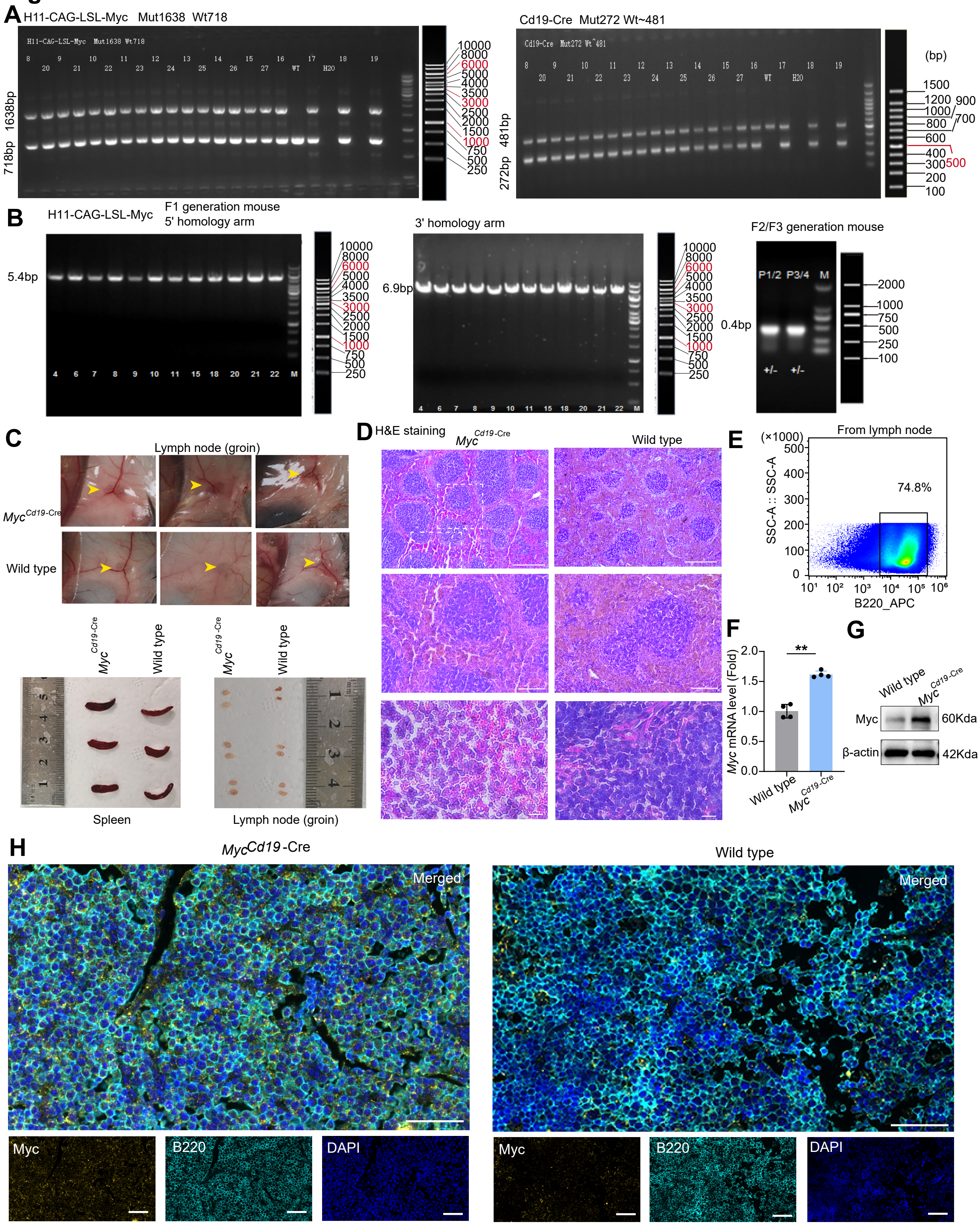

A
H11-CAG-LSL-Myc Mut1638 Wt718
Cd19-Cre Mut272 Wt~481
1638bp
718bp
481bp
272bp
(bp)
B
H11-CAG-LSL-Myc
F1 generation mouse
5' homology arm
3' homology arm
F2/F3 generation mouse
5.4bp
6.9bp
0.4bp
P1/2 P3/4 M
+/- +/-
C
Lymph node (groin)
Myc Cd19-Cre
Wild type
Spleen
Lymph node (groin)
D H&E staining
Myc Cd19-Cre
Wild type
E
From lymph node
74.8%
SSC-A :: SSC-A
B220_APC
F
**
Myc mRNA level (Fold)
Wild type
Myc Cd19-Cre
G
Wild type
Myc Cd19-Cre
Myc
60Kda
β-actin
42Kda
H
MycCd19-Cre
Wild type
Merged
Myc
B220
DAPI
