# Supplementary figures and images for: SOX5 Orchestrates Malignant Evolution via Promoter‐Centric Chromatin Remodeling in MYC‐Driven B‐Cell Lymphoma
Source: Adv Sci (Weinh). 2026 Jul 17:e76656. Online ahead of print. doi: 10.1002/advs.76656 (PMC13379260; doi:10.1002/advs.76656)

**Figure S2**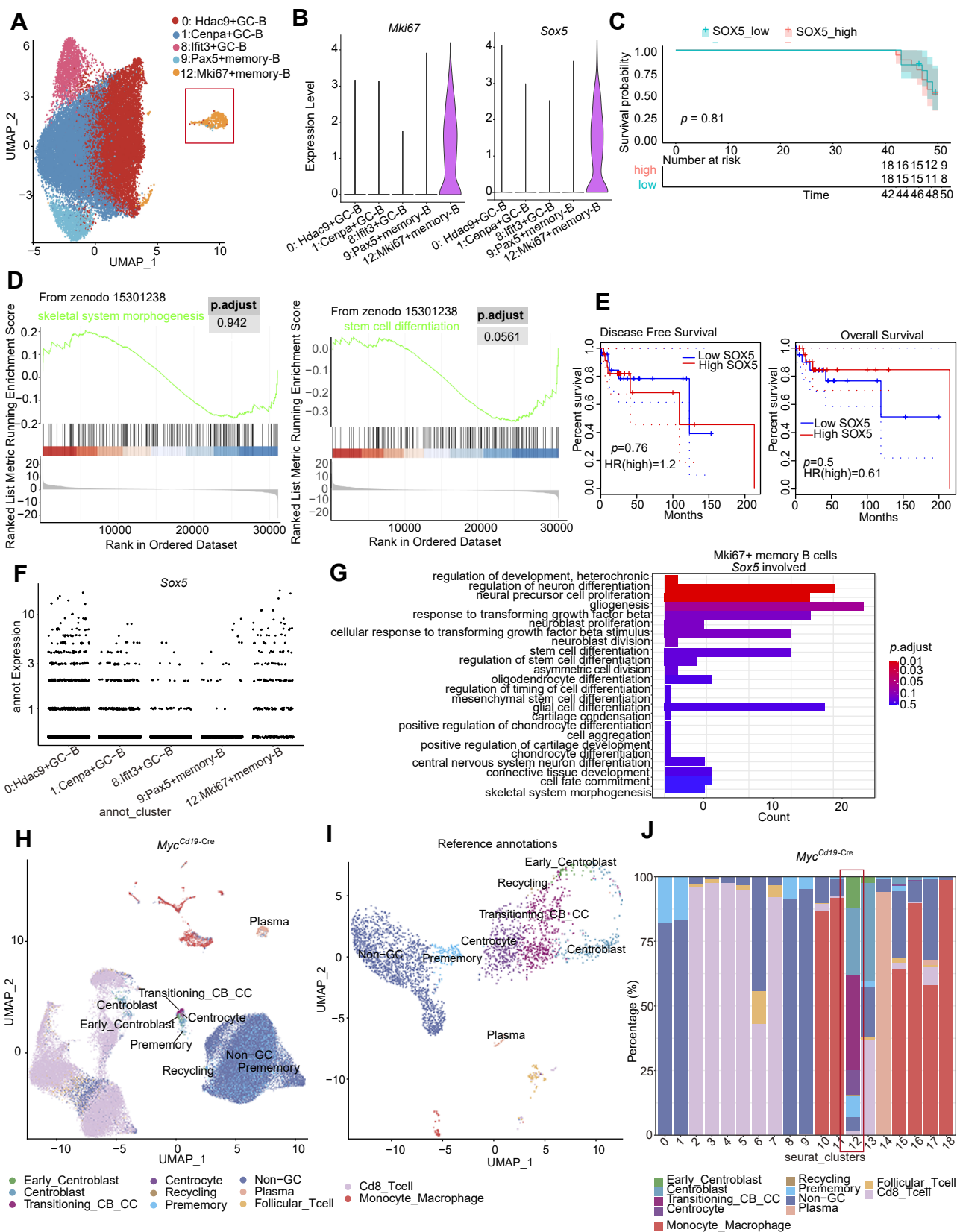

Supplement: Supplementary file 2 — Supporting file 2: advs76656‐sup‐0002‐FigureS1–S8.zip. [file ADVS-9999-e76656-s003.zip › Figure S2.pdf]

**Figure S3**

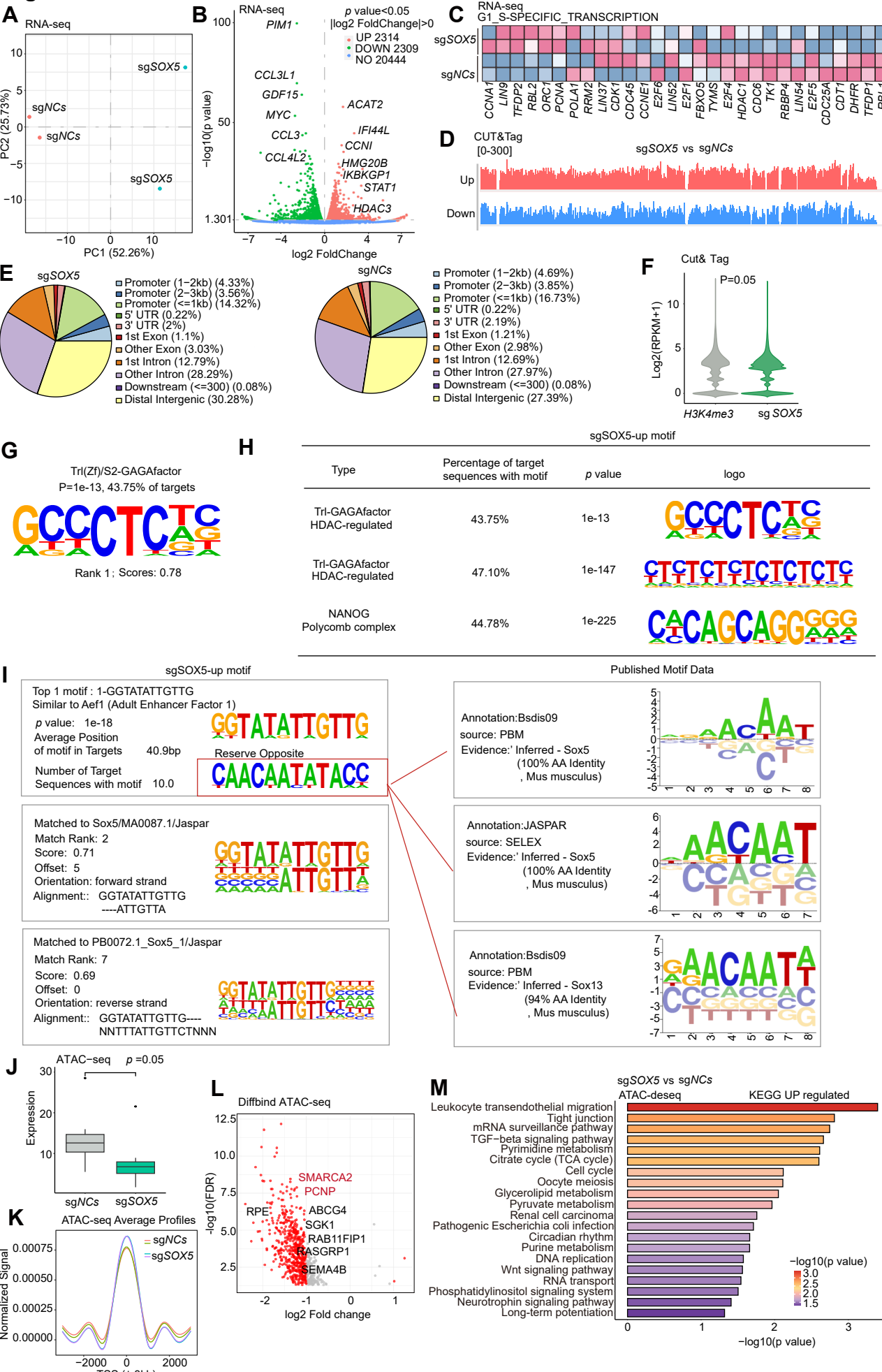

Supplement: Supplementary file 2 — Supporting file 2: advs76656‐sup‐0002‐FigureS1–S8.zip. [file ADVS-9999-e76656-s003.zip › Figure S3.pdf]

**Figure S4**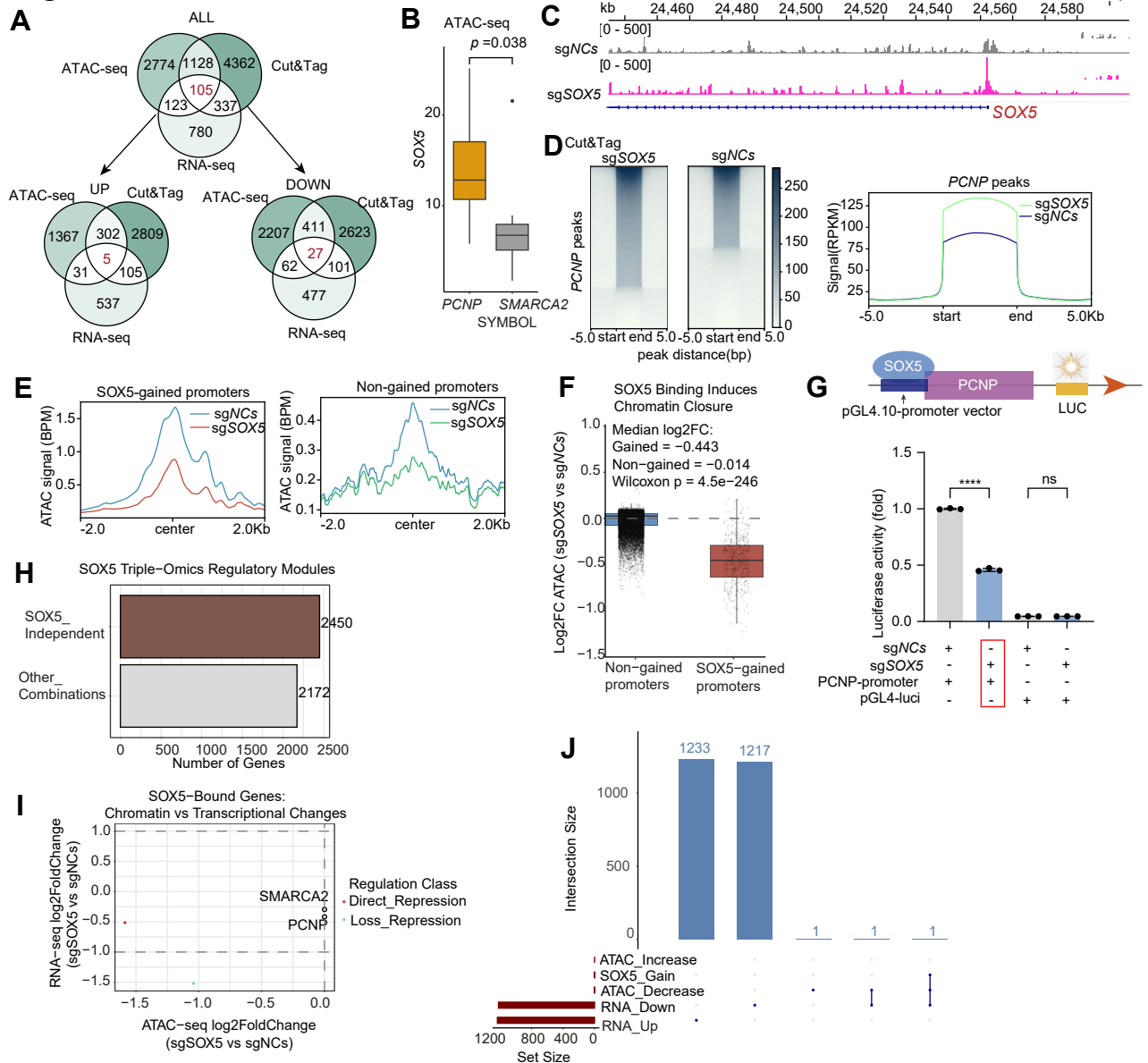

Supplement: Supplementary file 2 — Supporting file 2: advs76656‐sup‐0002‐FigureS1–S8.zip. [file ADVS-9999-e76656-s003.zip › Figure S4.pdf]

# Figure S5

## A

Merged/SOX5/PCNP/DAPI

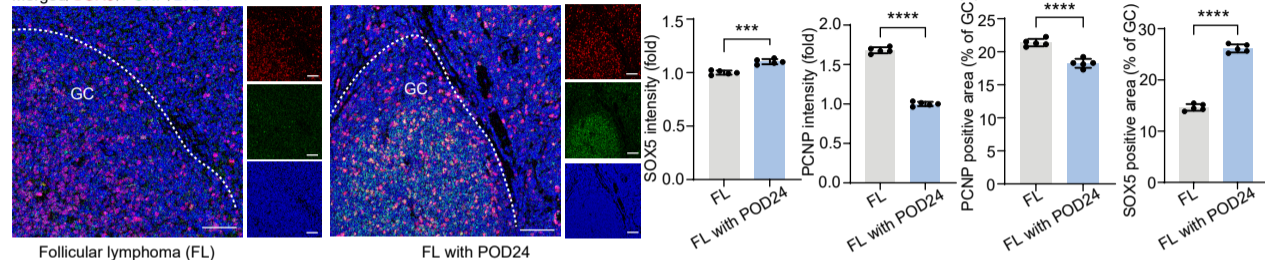

## B

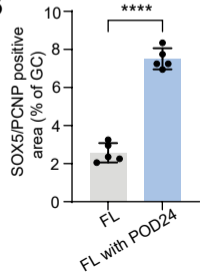

## C

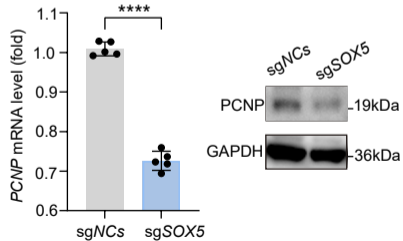

## D

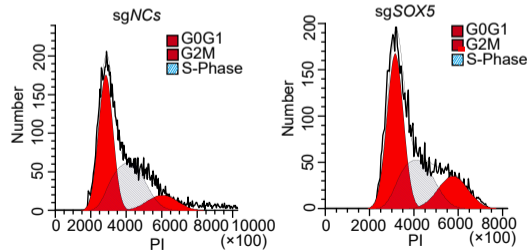

Supplement: Supplementary file 2 — Supporting file 2: advs76656‐sup‐0002‐FigureS1–S8.zip. [file ADVS-9999-e76656-s003.zip › Figure S5.pdf]

### Figure S6

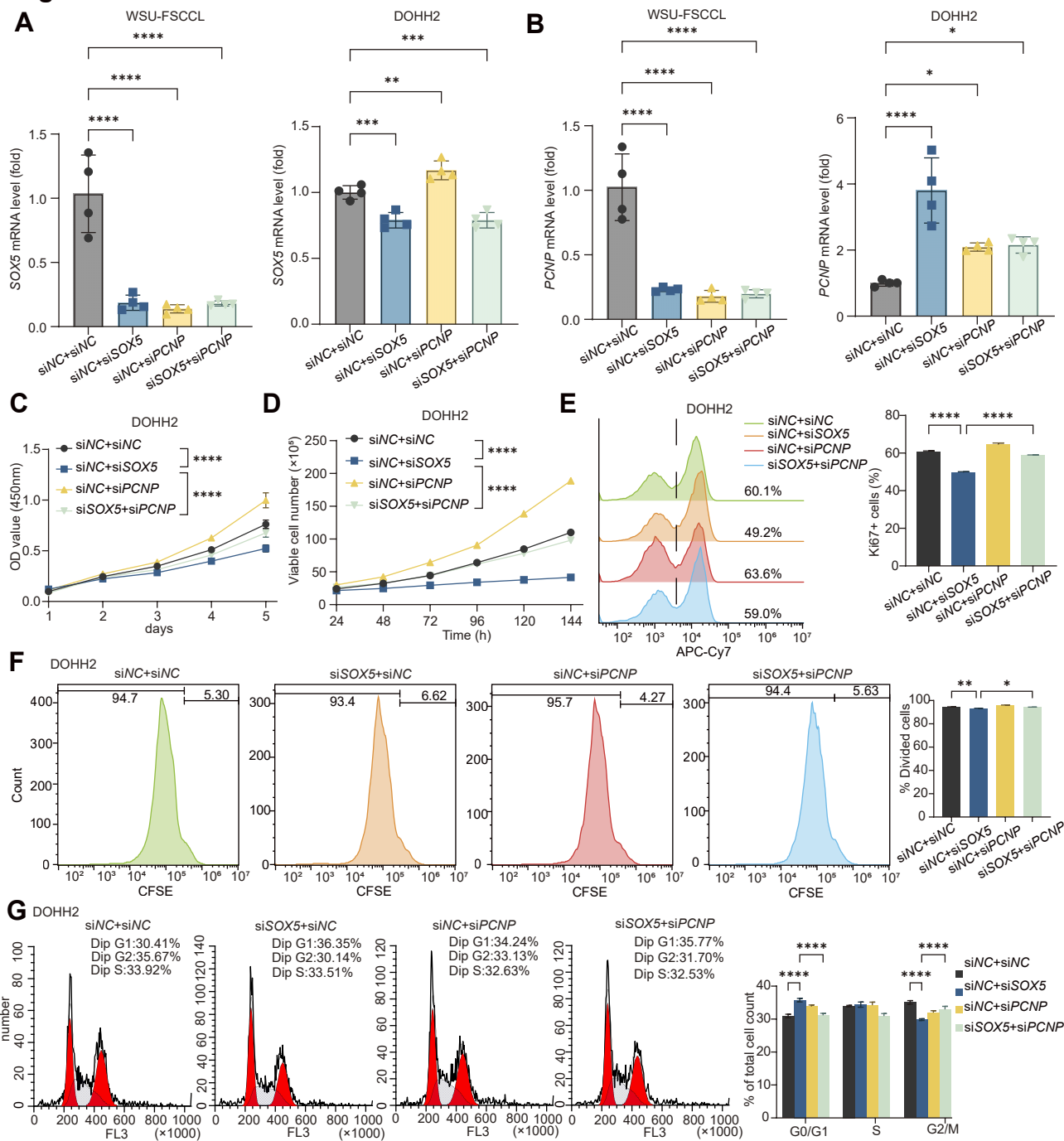

Supplement: Supplementary file 2 — Supporting file 2: advs76656‐sup‐0002‐FigureS1–S8.zip. [file ADVS-9999-e76656-s003.zip › Figure S6.pdf]

**Figure S7**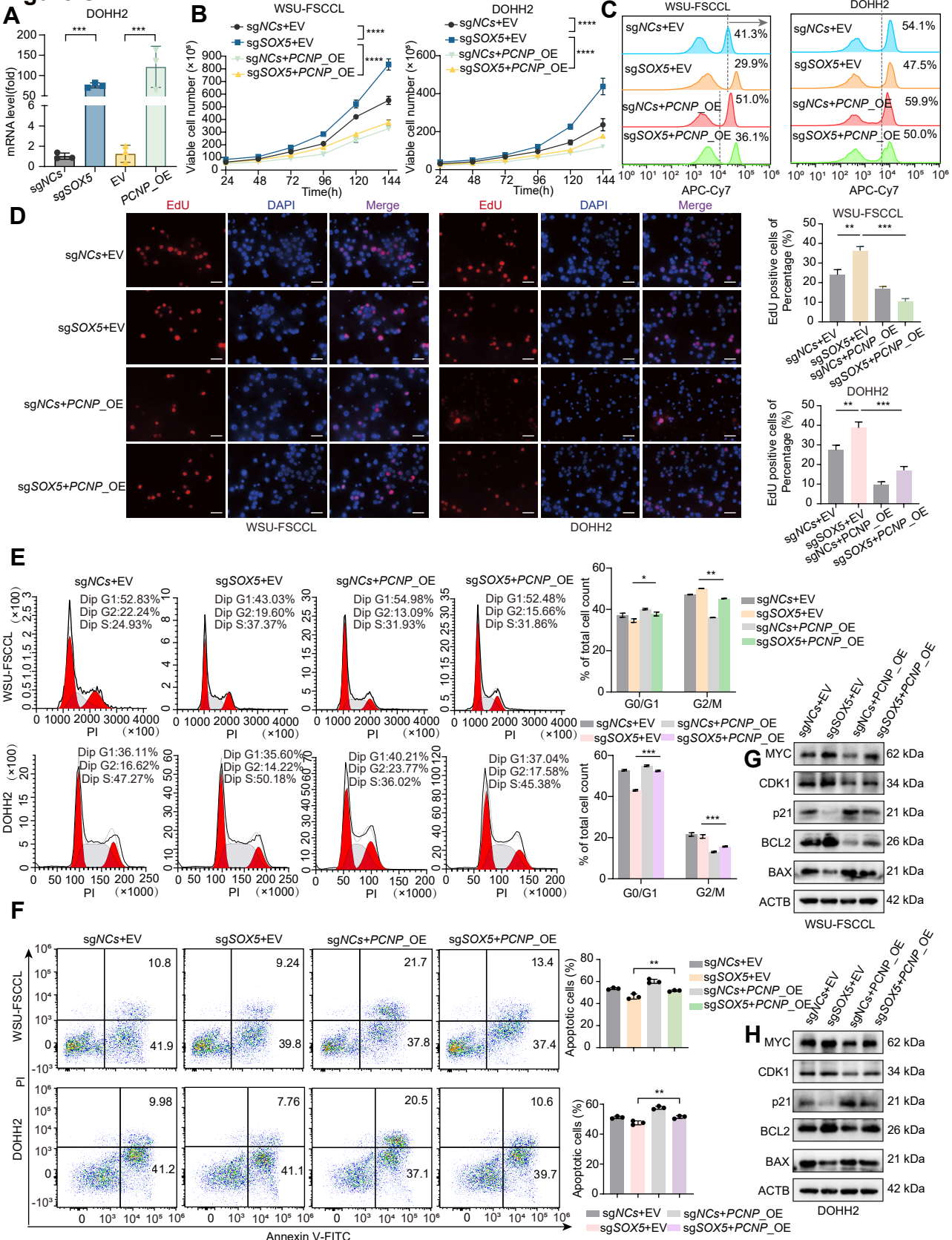

Supplement: Supplementary file 2 — Supporting file 2: advs76656‐sup‐0002‐FigureS1–S8.zip. [file ADVS-9999-e76656-s003.zip › Figure S7.pdf]

**Figure S8**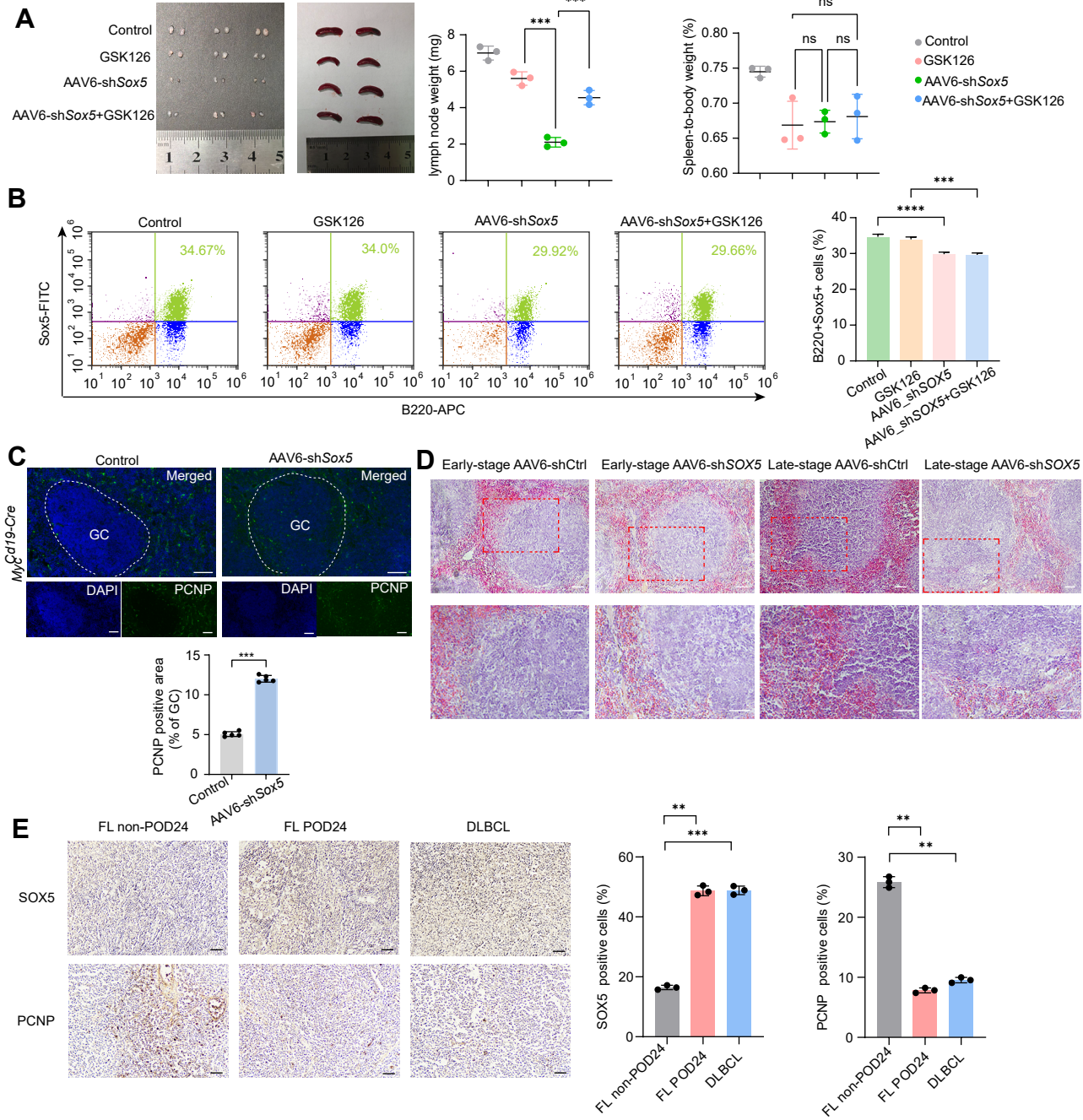

Supplement: Supplementary file 2 — Supporting file 2: advs76656‐sup‐0002‐FigureS1–S8.zip. [file ADVS-9999-e76656-s003.zip › Figure S8.pdf]
