# Supplementary material for: SOX5 Orchestrates Malignant Evolution via Promoter‐Centric Chromatin Remodeling in MYC‐Driven B‐Cell Lymphoma
Source: Adv Sci (Weinh). 2026 Jul 17:e76656. Online ahead of print. doi: 10.1002/advs.76656 (PMC13379260; doi:10.1002/advs.76656)
Supplement: Supplementary file 3 — Supporting file 3: advs76656‐sup‐0003‐TableS1–S6.zip. [file ADVS-9999-e76656-s002.zip › Table S3.docx]

**Table S3. Cluster 1 gene panel related to figure 3Q (top 30).**

|  | log2(fpkm+1) | | | |  |
| --- | --- | --- | --- | --- | --- |
| gene | sgSOX5-1 | sgSOX5-2 | NC-1 | NC-2 | gene_biotype |
| PIM1 | -1.17836286 | -1.246544248 | 1.22494791079336 | 1.19995919709759 | protein_coding |
| CCL3L1 | -1.162123583 | -0.907973574 | 1.12706006370314 | 0.943037092752977 | protein_coding |
| GDF15 | -1.309462099 | -1.153501076 | 1.19011720813286 | 1.27284596701738 | protein_coding |
| METRNL | -1.277689652 | -1.047419425 | 1.06899967104525 | 1.25610940642693 | protein_coding |
| GDAP1L1 | -1.007844775 | -0.778533383 | 1.06727501929087 | 0.719103138006759 | protein_coding |
| ROCK1P1 | -0.708408972 | -0.716017862 | 0.773959225422053 | 0.650467609149791 | protein_coding |
| TET3 | -0.463746095 | -0.607912472 | 0.55806984850491 | 0.513588718723646 | protein_coding |
| NFKBID | -0.633972443 | -0.779872095 | 0.660129434545519 | 0.753715103396953 | protein_coding |
| NIBAN3 | -0.377999097 | -0.45017633 | 0.339467177389169 | 0.488708250349421 | protein_coding |
| TIMM13 | -0.555674483 | -0.316742983 | 0.473496810442083 | 0.398920655274324 | protein_coding |
| TCEA1 | -0.357713596 | -0.320115971 | 0.385015016468087 | 0.292814550981386 | protein_coding |
| SH3BP2 | -0.510847334 | -0.303544291 | 0.313792077016172 | 0.500599548260432 | protein_coding |
| BTG1 | -0.292806576 | -0.303776611 | 0.369015921187067 | 0.22756726509631 | protein_coding |
| PDCD4 | -0.269023699 | -0.316167952 | 0.256847230570962 | 0.328344420166417 | protein_coding |
| CCAR1 | -0.236206982 | -0.321622528 | 0.198671062610627 | 0.359158447251938 | protein_coding |
| TRRAP | -0.178757883 | -0.317908151 | 0.21288257190332 | 0.283783462043037 | protein_coding |
| CDC25B | -0.195637889 | -0.310465884 | 0.236189015807331 | 0.269914757433614 | protein_coding |
| GFI1B | -0.203695878 | -0.580889605 | 0.324222096542728 | 0.460363386667799 | protein_coding |
| CASP8 | -0.286376667 | -0.142295517 | 0.238751831103768 | 0.189920352850875 | protein_coding |
| PAK2 | -0.21159113 | -0.166507515 | 0.195668343131961 | 0.182430302130694 | protein_coding |
| TP53RK | -0.250241983 | -0.230597036 | 0.397874099168087 | 0.0829649204292378 | protein_coding |
| BCL6 | -0.071206121 | -0.219535487 | 0.103990974848291 | 0.186750633034473 | protein_coding |
| BAX | -0.289532386 | -0.024259852 | 0.144230979897753 | 0.169561258144006 | protein_coding |
| ATG2B | -0.100825609 | -0.15681236 | 0.0779494306009632 | 0.179688538697666 | protein_coding |
| GAS7 | -0.085358391 | -0.085490559 | 0.054712792744034 | 0.116136157581647 | protein_coding |
| BAG6 | -0.075710305 | -0.157045709 | 0.103426722542758 | 0.129329292096228 | protein_coding |
| AUTS2 | -0.079736232 | -0.105307107 | 0.0268174799597813 | 0.15822585855147 | protein_coding |
| CPEB1 | -0.039568797 | -0.039568797 | 0.0236404453018599 | 0.0554971484864721 | protein_coding |
| GRPEL1 | -0.16903317 | -0.103774444 | 0.373546929756975 | -0.100739316 | protein_coding |
