# Supplementary material for: SOX5 Orchestrates Malignant Evolution via Promoter‐Centric Chromatin Remodeling in MYC‐Driven B‐Cell Lymphoma
Source: Adv Sci (Weinh). 2026 Jul 17:e76656. Online ahead of print. doi: 10.1002/advs.76656 (PMC13379260; doi:10.1002/advs.76656)
Supplement: Supplementary file 3 — Supporting file 3: advs76656‐sup‐0003‐TableS1–S6.zip. [file ADVS-9999-e76656-s002.zip › Table S4.docx]

**Table S4. Cluster 2 gene panel related to figure 3Q (top 30).**

|  | log2(fpkm+1) | | | |  |
| --- | --- | --- | --- | --- | --- |
| gene | sgSOX5-1 | sgSOX5-2 | NC-1 | NC-2 | gene_biotype |
| ALDOC | 0.838858515653841 | 0.811871438212167 | -0.797998236 | -0.852731717 | protein_coding |
| SLCO2B1 | 0.99168705129261 | 0.800594027697535 | -0.929162239 | -0.86311884 | protein_coding |
| NQO1 | 0.773283931521513 | 0.719886564466749 | -0.698644972 | -0.794525524 | protein_coding |
| MVD | 0.753285010168763 | 0.878793324881853 | -0.954197778 | -0.677880557 | protein_coding |
| ERG28 | 0.574396666678025 | 0.73121177030819 | -0.559606838 | -0.746001599 | protein_coding |
| ACAT2 | 0.714858721647115 | 0.631519799888647 | -0.759295361 | -0.587083161 | protein_coding |
| IFI44L | 0.728609060996737 | 0.616867260355822 | -0.626495542 | -0.71898078 | protein_coding |
| RSAD1 | 0.702197785313226 | 0.79308028364777 | -0.747613122 | -0.747664947 | protein_coding |
| DHCR7 | 0.613191026411654 | 0.512838422246032 | -0.524056319 | -0.601973129 | protein_coding |
| NFS1 | 0.541295780164216 | 0.648190383217308 | -0.610082939 | -0.579403224 | protein_coding |
| TM7SF2 | 0.686446298752285 | 0.709509169655961 | -0.737743294 | -0.658212175 | protein_coding |
| IDH1 | 0.454952282876586 | 0.503645125158356 | -0.525559513 | -0.433037895 | protein_coding |
| SCD | 0.448616056821868 | 0.43502716137569 | -0.484390128 | -0.399253091 | protein_coding |
| CCNI | 0.437043846737464 | 0.569121579886469 | -0.378911562 | -0.627253865 | protein_coding |
| SLC12A8 | 0.455446020689457 | 0.456896052072849 | -0.522570344 | -0.389771729 | protein_coding |
| LSS | 0.624393905679291 | 0.40930112870597 | -0.650246608 | -0.383448426 | protein_coding |
| REC8 | 0.414193215795096 | 0.494919741635715 | -0.491655609 | -0.417457348 | protein_coding |
| ALOX5 | 0.460643876000539 | 0.470006476932287 | -0.519445897 | -0.411204456 | protein_coding |
| FTH1 | 0.372979071563481 | 0.388762405568208 | -0.367190292 | -0.394551185 | protein_coding |
| PCYOX1L | 0.558442695871423 | 0.64014435614676 | -0.744562721 | -0.454024331 | protein_coding |
| ABHD17A | 0.487309601413003 | 0.501185786258099 | -0.551553716 | -0.436941672 | protein_coding |
| MVK | 0.4301573954564 | 0.477320547570791 | -0.50158167 | -0.405896273 | protein_coding |
| FDPS | 0.436126355326195 | 0.389914433754581 | -0.41872814 | -0.407312649 | protein_coding |
| PKM | 0.403588032801774 | 0.380952304430399 | -0.447550636 | -0.336989701 | protein_coding |
| AK8 | 0.655123022699337 | 1.29400043633112 | -1.075882223 | -0.873241236 | protein_coding |
| PRKCB | 0.366036284341345 | 0.409314284916465 | -0.341059667 | -0.434290902 | protein_coding |
| AP2S1 | 0.579760002131419 | 0.568260219125162 | -0.701873164 | -0.446147057 | protein_coding |
| BCAT2 | 0.37748724167369 | 0.495948956151305 | -0.38033705 | -0.493099148 | protein_coding |
| PAPSS1 | 0.423118678074315 | 0.491236792248755 | -0.46975471 | -0.44460076 | protein_coding |
